# A Review of the Potential Use of Antioxidants in Spinal Cord Injuries

**DOI:** 10.3390/antiox14091081

**Published:** 2025-09-03

**Authors:** Agnieszka Nowacka, Maciej Śniegocki, Ewa Ziółkowska

**Affiliations:** 1 Department of Neurosurgery, Nicolaus Copernicus University in Toruń, Collegium Medicum in Bydgoszcz, ul. Curie Skłodowskiej 9, 85-094 Bydgoszcz, Poland; 2 Department of Pediatrics, School of Medicine, Washington University in St. Louis, St. Louis, MO 63110, USA

**Keywords:** spinal cord injury, SCI, oxidative stress, antioxidants, antioxidant therapy, natural compounds

## Abstract

Spinal cord injury (SCI) is a debilitating neurological condition marked by primary mechanical damage followed by a complex secondary injury cascade, in which oxidative stress plays a central role. Mitochondrial dysfunction, ionic imbalance, and inflammatory responses drive excessive generation of reactive oxygen and nitrogen species, leading to lipid peroxidation, protein and DNA damage, apoptosis, and progressive neurological impairment. Antioxidant-based therapies have emerged as promising neuroprotective strategies, with compounds such as A91 peptide, curcumin, edaravone, ginsenosides, and glutathione demonstrating preclinical efficacy in reducing oxidative damage, restoring redox balance, modulating signaling pathways (e.g., Nrf2, NF-κB, MAPK, PI3K/Akt), and enhancing neuronal survival. While therapeutic outcomes depend on injury severity, timing, and combinatorial approaches, translating these findings into clinical practice and integrating antioxidants with cell-based therapies, biomaterials, and rehabilitation offers a critical avenue for improving functional recovery in SCI.

## 1. Introduction

Spinal cord injury (SCI) represents one of the most devastating neurological conditions, with profound implications for patient quality of life and significant socioeconomic burden worldwide [1,2]. The global incidence of SCI varies considerably by region, ranging from 7 to 37 cases per 100,000 individuals annually, with traffic accidents constituting the leading cause, followed by falls, violence, and sports/recreation accidents [3,4,5,6]. Demographically, the average age at injury is 42 years, with males comprising 80% of cases, and incomplete tetraplegia being the most frequent injury type, followed by incomplete paraplegia, complete paraplegia, and complete tetraplegia [3,4,7,8]. Tragically, less than 1% of patients experience complete recovery by hospital discharge, underscoring the critical need for innovative therapeutic interventions [3,4,7,8].

**Figure 1:**
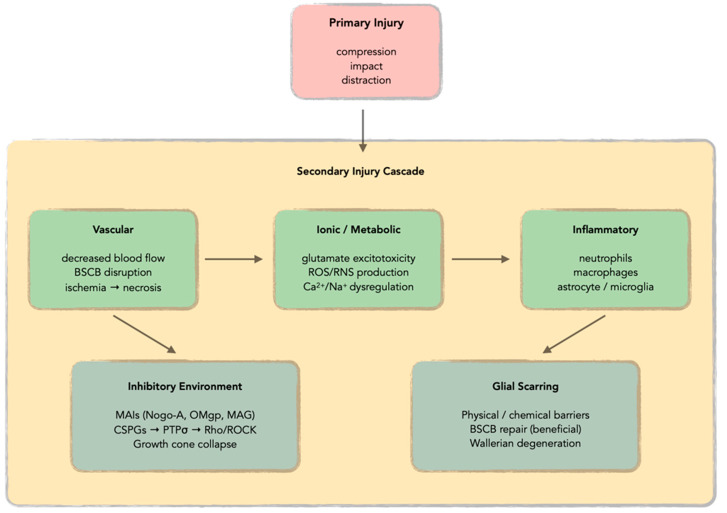
Spinal cord injury.

The pathophysiology of SCI involves a complex biphasic process consisting of primary and secondary injury mechanisms (Figure 1) [9,10,11,12,13,14]. Primary injury occurs through various mechanical forces, including compression, impact, distraction, and laceration/transection, which directly damage neurons, glial cells, and the neurovasculature of the spinal cord [9,10,11,12]. The extent and level of primary injury largely determine the severity and outcome of SCI [9,10,12]. However, it is the secondary injury cascade that represents the primary therapeutic target, as it evolves over hours to weeks following the initial trauma and significantly contributes to the final neurological deficit [9,10,11,12,13]. This secondary injury cascade encompasses multiple interconnected pathological processes, including vascular alterations, ionic disruption, metabolic dysfunction, neuroinflammation, creation of an inhibitory environment, and scar formation [9,10,11,12,13].

Vascular dysfunction represents one of the earliest secondary injury mechanisms, with decreased blood flow leading to ischemia and subsequent cellular necrosis due to oxygen and glucose deprivation [15,16,17]. Concurrently, disruption of the blood–spinal cord barrier (BSCB) promotes infiltration of immune mediators, leading to edema and exacerbation of the pro-inflammatory environment [18,19,20,21,22,23]. Ionic dysregulation, particularly involving excessive glutamate release and subsequent over-activation of NMDA and AMPA receptors, results in increased sodium and calcium influx, making neurons and oligodendrocytes vulnerable to cell death [24,25,26,27,28]. This glutamate excitotoxicity disturbs ionic homeostasis and mitochondrial function, ultimately resulting in axonal demyelination and neuronal loss at the injury site [25,27,29,30,31,32].

The metabolic consequences of SCI involve the production of reactive oxygen species (ROS) and reactive nitrogen species (RNS), which react with polyunsaturated fatty acids of cellular membranes, leading to lipid peroxidation and damage at protein and nucleic acid levels [33,34,35,36]. These free radicals also cause architectural alterations of the cytoskeleton and organelle membranes, mitochondrial dysfunction, and increased intracellular calcium uptake [37,38]. The inflammatory response following SCI is particularly complex, involving both resident cells (astrocytes, microglia) and infiltrating peripheral immune cells [39,40,41]. The disruption of the BSCB facilitates the invasion of non-resident cells that chronically persist within the spinal cord [18,21]. Neutrophils are the first infiltrating immune cells, peaking around 1 day post-injury in both rodents and humans, followed by macrophages that contribute to both tissue damage and repair processes [42,43,44,45].

A critical aspect of SCI pathophysiology is the establishment of an inhibitory environment that prevents axonal regeneration [46,47,48,49,50]. The extracellular matrix (ECM) surrounding CNS cells becomes populated with inhibitory molecules following injury, including myelin-associated inhibitors (MAIs) such as Nogo-A, oligodendrocyte myelin glycoprotein (OMgp), and myelin-associated glycoprotein (MAG) [51,52,53]. These molecules have been demonstrated to potently inhibit neurite outgrowth and induce growth cone collapse in various experimental models [51,52,53,54]. Additionally, chondroitin sulfate proteoglycans (CSPGs) are actively secreted into the ECM, primarily by reactive astrocytes, creating an abundance of inhibitory molecules at the injury site [55,56,57,58]. The inhibitory effect of CSPGs is mediated through the protein tyrosine phosphatase sigma (PTPσ) receptor, which activates the GTPase Rho/ROCK signaling pathway, leading to axonal growth inhibition and dystrophic growth cone formation [59,60,61].

The formation of glial scarring represents another major impediment to recovery, involving the deposition of inhibitory ECM components and creating both chemical and physical barriers to axonal regeneration [47,62]. However, recent research has revealed the dual nature of glial scarring, with beneficial effects including repair of the BSCB, restraint of inflammatory responses, and sequestration of toxic species to the injury site [63,64,65]. The chronic phase of SCI is characterized by ongoing Wallerian degeneration of injured axons, which may take years for complete removal, and potential syrinx formation that can cause additional neurological deterioration [66,67,68,69].

Current clinical management of acute SCI focuses primarily on early surgical decompression within 24 h, as recommended by recent AOSpine guidelines and the American Association of Neurological Surgeons [70,71]. Hemodynamic monitoring and maintenance of mean arterial blood pressure between 85 and 90 mmHg are also crucial during the acute phase [72]. The use of methylprednisolone sodium succinate (MPSS), once a standard treatment based on the NASCIS trials, is no longer recommended due to increased complication risks, including pneumonia, gastrointestinal hemorrhage, and sepsis [73,74].

Innovative therapeutic approaches currently under investigation span multiple domains, including molecular therapies, cell-based strategies, biomaterial applications, and novel neurorehabilitation protocols [75,76,77]. Molecular therapies being explored include neuroprotective agents such as riluzole, which has shown promise in phase I and II clinical trials for improving motor outcomes in SCI patients [78,79,80,81,82,83]. Hormonal therapies, including progesterone and estrogen, have demonstrated neuroprotective effects in preclinical models through anti-inflammatory mechanisms and promotion of axonal regeneration [84,85]. Other promising molecular approaches include the use of resveratrol, which targets multiple pathways, including SIRT1/AMPK signaling, to improve motor function recovery, and omega-3 polyunsaturated fatty acids, which modulate inflammatory responses and promote functional recovery [86,87,88].

Cell-based therapies represent another major therapeutic avenue, with various cell types being investigated, including embryonic stem cells, induced pluripotent stem cells (iPSCs), neural stem cells, Schwann cells, olfactory ensheathing cells, and mesenchymal stem cells [89,90]. Clinical trials have demonstrated the safety and feasibility of these approaches, with some showing modest functional improvements [89,91]. Biomaterial strategies focus on providing scaffolds for tissue repair and cell delivery, with hydrogels being particularly promising due to their compatibility with nervous tissue properties [75,76,77,92,93,94].

Perhaps most remarkably, recent advances in neurorehabilitation have revolutionized treatment possibilities through epidural electrical stimulation (EES) combined with intensive training [95,96,97]. Groundbreaking clinical studies have demonstrated the restoration of independent walking in patients with chronic complete spinal cord injury through targeted neurotechnology that modulates lumbosacral spinal networks [98,99]. The success of these interventions highlights the remarkable plasticity of spinal neural networks and the potential for functional recovery even in chronic, complete injuries [95,97,98,99,100,101].

This review critically examines the role of oxidative stress in spinal cord injury (SCI) and consolidates current evidence on the therapeutic potential of antioxidant-based interventions to attenuate secondary injury and enhance neuroprotection. It highlights the contribution of excessive reactive oxygen and nitrogen species to lipid peroxidation, protein and DNA oxidation, mitochondrial dysfunction, apoptosis, and impaired functional recovery. This review further explores the underlying mechanisms of oxidative damage and provides a comprehensive overview of natural and synthetic antioxidants—including curcumin, edaravone, ginsenosides, and glutathione—that have demonstrated neuroprotective, anti-inflammatory, and antioxidative effects in preclinical models of SCI. Finally, it discusses future perspectives, emphasizing the importance of tailoring antioxidant therapies to injury severity and temporal phase, as well as the potential of combination approaches with stem cells or biomaterials to optimize functional recovery.

## 2. Mechanisms of Oxidative Stress in Spinal Cord Injury

### 2.1. Mitochondrial Dysfunction and Primary ROS Generation

The molecular mechanisms underlying oxidative stress in spinal cord injury involve complex, interconnected pathways that begin immediately after the initial trauma and continue to propagate damage through secondary injury cascades (Table 1) [36,102,103]. The primary sources of reactive oxygen species generation in the injured spinal cord are fundamentally rooted in mitochondrial dysfunction, where compromised energy metabolism leads to the formation of superoxide, hydroxyl radical, singlet oxygen, and hydrogen peroxide [104,105,106,107,108]. Superoxide, the one-electron reduction product of molecular oxygen, represents the initial ROS formed in this cascade, while hydrogen peroxide results from the two-electron transfer process [109,110,111]. The involvement of transition metal ions, particularly Fe^2+^ and Cu^+^, becomes critical in this process as they are capable of transferring electrons and catalyzing the formation of highly reactive hydroxyl radicals through Fenton chemistry [112,113]. Within the mitochondrial matrix, the electron transport chain becomes severely compromised following SCI, leading to electron leakage at complexes I and III, which directly reduces molecular oxygen to form superoxide anions [104,114,115]. This mitochondrial dysfunction creates a self-perpetuating cycle where ROS production impairs mitochondrial function, which in turn generates more ROS, establishing a destructive feedback loop that continues long after the initial injury [104,116,117,118].

### 2.2. Electron Transport Chain Disruption and Energy Crisis

The disruption of mitochondrial respiratory chain function following SCI occurs through multiple molecular mechanisms, including direct damage to electron transport proteins, depletion of key cofactors such as coenzyme Q10 and cytochrome c, and alterations in mitochondrial membrane potential that affect the efficiency of ATP synthesis [39,119,120]. The subsequent energy crisis created by impaired ATP production compromises cellular ion pumps, particularly Na^+^/K^+^-ATPase and Ca^2+^-ATPase, leading to ionic imbalances that further exacerbate oxidative stress through calcium-dependent activation of phospholipases, proteases, and endonucleases [121,122,123,124]. Calcium overload within mitochondria triggers the formation of the mitochondrial permeability transition pore, a critical event that releases cytochrome c and other pro-apoptotic factors into the cytoplasm while simultaneously increasing ROS production and depleting cellular energy reserves [125,126,127,128]. The formation of peroxynitrite (ONOO^−^) through the diffusion-limited combination of nitric oxide and superoxide radicals represents another crucial mechanism of oxidative damage, as this highly reactive species can nitrosylate proteins, oxidize lipids, and damage DNA at rates approaching diffusion limits [129,130,131].

### 2.3. Lipid Peroxidation and Membrane Damage

Lipid peroxidation emerges as one of the most destructive consequences of ROS generation in SCI, initiated primarily by hydroxyl radicals that abstract hydrogen atoms from polyunsaturated fatty acids in cellular membranes, creating lipid radicals that propagate chain reactions of membrane damage [132,133]. This process is particularly devastating in neural tissue due to the high concentration of polyunsaturated fatty acids in neuronal and glial cell membranes, leading to the formation of toxic aldehydes such as 4-hydroxynonenal and malondialdehyde that can covalently modify proteins and further disrupt cellular function [132,134,135,136,137]. The iron-catalyzed nature of lipid peroxidation becomes especially relevant in SCI, as tissue injury releases iron from hemoglobin, ferritin, and other iron-containing proteins, providing the catalytic metal necessary for Fenton reactions that generate hydroxyl radicals from hydrogen peroxide [112,113,138,139,140]. The breakdown of the blood–spinal cord barrier following injury allows infiltration of iron-rich blood cells and plasma proteins, further contributing to the iron burden and oxidative stress within the injured tissue [21,141,142,143].

### 2.4. Cellular Sources of ROS Production

The cellular sources of ROS production in SCI extend beyond mitochondria to include activated microglia and infiltrating macrophages, which generate superoxide and other reactive species through NADPH oxidase activation as part of the inflammatory response [40,144,145,146,147,148,149,150]. The dual role of microglia in both neuroprotection and neurodegeneration becomes apparent through their ROS production patterns, where controlled ROS generation can serve signaling functions, but excessive production leads to tissue damage and propagation of secondary injury [40,144,145,146,148,149,151,152,153]. Neutrophils that infiltrate the injury site within hours of trauma contribute significantly to oxidative stress through myeloperoxidase-mediated production of hypochlorous acid and other chlorinated oxidants, as well as through the respiratory burst that generates massive amounts of superoxide and hydrogen peroxide [154,155,156,157]. The xanthine oxidase pathway becomes activated following SCI due to ischemia–reperfusion injury, converting from its normal dehydrogenase form to the oxidase form that produces superoxide during the metabolism of hypoxanthine and xanthine derived from ATP breakdown [158,159,160,161].

### 2.5. Protein Oxidation and Functional Impairment

Protein oxidation represents another fundamental mechanism of oxidative damage in SCI, where ROS directly modify amino acid residues, leading to protein aggregation, loss of enzymatic activity, and disruption of protein–protein interactions essential for cellular function [162,163,164]. The oxidation of critical antioxidant enzymes, including superoxide dismutase, catalase, and glutathione peroxidase, creates a vicious cycle where the cellular defense mechanisms against oxidative stress become compromised, allowing ROS levels to increase further [165,166,167]. Cysteine residues in proteins are particularly vulnerable to oxidation, forming disulfide bonds that can alter protein conformation and function, while methionine residues can be oxidized to methionine sulfoxide, affecting protein stability and activity [168,169,170,171,172,173]. The carbonylation of proteins through reaction with aldehydes derived from lipid peroxidation creates irreversible modifications that mark proteins for degradation and contribute to the overall cellular dysfunction observed in SCI [168,174,175].

### 2.6. DNA Oxidation and Genomic Instability

DNA oxidation mechanisms in SCI involve direct attack by hydroxyl radicals on DNA bases, particularly guanine, to form 8-hydroxyguanosine, as well as strand breaks caused by ROS-mediated sugar–phosphate backbone damage [176,177,178,179,180]. The repair of oxidative DNA damage requires significant cellular energy expenditure and can lead to mutations if repair mechanisms are overwhelmed, contributing to genomic instability and potential activation of cell death pathways [181,182,183]. Mitochondrial DNA is particularly susceptible to oxidative damage due to its proximity to ROS-generating sites in the electron transport chain and the limited DNA repair mechanisms available in mitochondria compared to nuclear DNA [184,185,186,187]. The accumulation of mitochondrial DNA mutations can further impair electron transport chain function, creating additional sources of ROS and perpetuating the cycle of oxidative damage [188,189,190,191,192].

### 2.7. Antioxidant System Depletion and Defense Mechanisms

The depletion of endogenous antioxidant systems represents a critical aspect of oxidative stress mechanisms in SCI, where the consumption of glutathione, ascorbic acid, α-tocopherol, and other non-enzymatic antioxidants occurs rapidly following injury, while the activities of antioxidant enzymes become compromised through direct oxidative modification or transcriptional downregulation [34,193,194,195]. The glutathione system, consisting of reduced glutathione (GSH), glutathione peroxidase, glutathione reductase, and NADPH, becomes particularly depleted in SCI as it serves as the primary defense against hydrogen peroxide and lipid peroxides [34,36,196,197,198]. The recycling of oxidized glutathione back to its reduced form requires NADPH, which becomes limited due to impaired glucose metabolism and pentose phosphate pathway dysfunction following injury, further compromising antioxidant capacity [199,200,201,202,203,204]. The transcriptional regulation of antioxidant enzyme expression through the Nrf2-ARE pathway becomes disrupted in SCI, leading to decreased synthesis of protective enzymes at a time when oxidative stress is maximal [205,206,207,208].

### 2.8. Signal Transduction Pathways and Oxidative Stress Response

Signal transduction pathways activated by oxidative stress in SCI include the c-Jun N-terminal kinase (JNK) pathway, which responds to ROS accumulation and mitochondrial dysfunction by promoting apoptotic cell death through phosphorylation of pro-apoptotic proteins and transcription factors [103,209]. The p38 MAPK pathway also becomes activated by oxidative stress, leading to inflammatory gene expression and further propagation of tissue damage through cytokine production and additional ROS generation [210,211,212,213]. Nuclear factor-κB (NF-κB) activation by ROS leads to transcription of inflammatory mediators while simultaneously providing some protective effects through upregulation of antioxidant genes, demonstrating the complex dual nature of oxidative stress signaling [214,215,216,217]. The activation of poly(ADP-ribose) polymerase (PARP) by DNA damage consumes cellular NAD+ and ATP, contributing to energy depletion and cell death, while the excessive PARP activation can itself become a source of additional oxidative stress through depletion of cellular energy reserves [218,219,220,221,222,223,224]. These interconnected mechanisms of oxidative stress in SCI create a complex pathophysiological environment where multiple sources of ROS generation, compromised antioxidant defenses, and oxidative damage to cellular components combine to propagate secondary injury and limit the potential for functional recovery [66,144,145,225,226,227,228].

## 3. Antioxidants in Spinal Cord Injuries

Given the central role of oxidative stress in SCI pathophysiology, therapeutic interventions targeting this mechanism through exogenous antioxidant administration have emerged as promising neuroprotective strategies with the potential to limit secondary injury progression and improve functional outcomes (Table 2).

### 3.1. A91 Peptide

The A91 peptide represents a therapeutic candidate for spinal cord injury management, demonstrating considerable potential through its immunomodulatory properties and neuroprotective mechanisms [229,230]. Derived from myelin basic protein, this peptide exhibits anti-inflammatory effects, attenuates apoptotic processes, and upregulates neurotrophic factor production, thereby facilitating enhanced functional outcomes following spinal cord trauma [229,230,231].

Research by Garcia et al. investigating A91 peptide immunization has yielded compelling evidence for its neuroprotective efficacy in spinal cord injury models, establishing a clear mechanistic framework for its therapeutic potential [230]. The findings demonstrate that A91 immunization significantly attenuates nitric oxide production both in controlled laboratory conditions and in living animal models [230]. In vitro studies revealed that neural-derived peptide immunization, including A91, substantially reduced nitric oxide synthesis by glial cells when exposed to autoreactive T cells, a finding that was corroborated by in vivo experiments showing decreased nitric oxide concentrations at spinal cord injury sites in immunized animals [230]. Furthermore, the neuroprotective effects extend beyond simple nitric oxide reduction, as A91 immunization was found to downregulate inducible nitric oxide synthase gene expression at the injury site, thereby targeting the enzymatic source of excessive nitric oxide production during inflammatory responses [230]. These findings collectively support the hypothesis that A91-mediated protective autoimmunity operates through the suppression of detrimental nitric oxide pathways, creating a microenvironment conducive to neuronal survival and tissue preservation [230]. By mitigating reactive nitrogen species and their associated neurodegenerative cascades, including lipid peroxidation, A91 immunization represents a promising therapeutic strategy that harnesses the body’s immune system to promote neuroprotection and functional recovery following spinal cord trauma [230].

Research on A91-pulsed dendritic cells (A91-DC) has revealed significant therapeutic potential for spinal cord injury treatment through enhanced neurotrophic factor modulation and neuroprotective mechanisms [232]. In vitro investigations demonstrated that A91-DC effectively stimulated T cell populations to increase their secretion of neurotrophic factors, indicating a direct immunomodulatory effect that promotes beneficial cellular responses [232]. These laboratory findings were substantiated through in vivo experimentation using spinal cord injury mouse models, where A91-DC vaccination resulted in elevated expression levels of critical neurotrophic factors, specifically brain-derived neurotrophic factor (BDNF) and neurotrophin-3 (NT-3), within the injured spinal cord tissue during the subacute injury phase [232]. Histological analysis further confirmed that A91-DC vaccination provided significant neuroprotective benefits beyond neurotrophic factor enhancement, demonstrating measurable tissue preservation and reduced injury-related damage [232]. These comprehensive findings establish A91-DC vaccination as a promising minimally invasive therapeutic approach that leverages the body’s immune system to deliver sustained neurotrophic support to the injured spinal cord [232].

The therapeutic efficacy of A91 peptide immunization in spinal cord injury demonstrates a critical dependency on injury severity, revealing disparate immunomodulatory profiles that fundamentally alter treatment outcomes [233]. In moderate spinal cord injury models, A91 immunization elicited a favorable gene expression profile characterized by significant downregulation of pro-inflammatory mediators, including IL-6, IL-1β, and TNF-α, while concurrently upregulating anti-inflammatory and neuroprotective factors, such as IL-10, IL-4, and IGF-1, with no discernible effects on IL-12 and IFN-γ expression [233]. This balanced immunomodulatory response in moderate injury conditions creates a microenvironmental shift toward neuroprotection, facilitating tissue preservation and functional recovery through the suppression of detrimental inflammatory cascades and enhancement of reparative mechanisms [233]. Conversely, severe spinal cord contusion injury fundamentally altered A91’s therapeutic profile, resulting in detrimental outcomes characterized by significant upregulation of pro-inflammatory genes, including IL-12, IL-1β, and IFN-γ, alongside increased IGF-1 expression, while anti-inflammatory markers IL-4 and IL-10 remained unchanged compared to controls [233]. This paradoxical response in severe injury contexts suggests that the overwhelming tissue damage and inflammatory burden may subvert A91’s typical neuroprotective mechanisms, potentially exacerbating the pro-inflammatory state and contributing to secondary injury processes [233]. These findings underscore the critical importance of injury severity stratification in developing A91-based therapeutic protocols and highlight the need for injury-specific treatment approaches that account for the complex interplay between peptide immunization and the varying pathophysiological environments encountered across the spectrum of spinal cord injury severity [233].

A91 immunization demonstrates robust and sustained neuroprotective effects following moderate spinal cord injury through enhanced neurotrophic factor production, sustained immune activation, and improved functional recovery, though these benefits are critically dependent on injury severity [231]. In the acute phase at 21 days post-injury, A91-immunized animals exhibited significantly elevated levels of brain-derived neurotrophic factor (BDNF) (0.165 ± 0.01 versus 0.076 ± 0.02 in controls, *p* = 0.002) and neurotrophin-3 (NT-3) (0.133 ± 0.02 versus 0.062 ± 0.01 in controls, *p* = 0.03) at the lesion site, establishing an early neuroprotective environment conducive to tissue preservation and repair [231]. This therapeutic benefit was sustained into the chronic phase at 4 months post-injury, where A91 immunization continued to promote neurotrophic factor production in moderate contusion and hemisection models, supported by a persistent T-cell response evidenced by a significantly elevated stimulation index (1.87 ± 0.09 versus 0.89 ± 0.05 in controls, *p* = 0.001) [231]. The molecular benefits translated into meaningful functional improvements, with A91-immunized animals demonstrating superior motor recovery scores from the first week (6.3 ± 0.2 versus 2.4 ± 0.3 in controls) to 4 months post-injury (10.8 ± 0.2 versus 8.1 ± 0.3 BBB scores at two months), indicating sustained neurological improvement [231]. However, these therapeutic effects were strictly limited to moderate injury paradigms, as severe contusion or complete transection models failed to demonstrate enhanced neurotrophic factor production or functional recovery following A91 immunization, suggesting that the severity-dependent immunodepression characteristic of severe spinal cord injuries may overwhelm the protective autoimmune mechanisms elicited by A91 treatment [231]. These findings establish A91 immunization as a promising therapeutic intervention for moderate spinal cord injuries while highlighting the critical importance of injury severity stratification in clinical translation and the need for alternative or combinatorial approaches for severe injury cases [231].

### 3.2. Allicin

Allicin, a bioactive compound sourced from garlic, exhibits potential neuroprotective properties relevant to spinal cord injuries [234]. It demonstrates comprehensive neuroprotective efficacy in traumatic spinal cord injury through multifaceted mechanisms that target both primary injury responses and secondary pathological cascades [234]. Treatment with allicin at doses of 5 or 10 mg/kg significantly enhanced functional recovery, as evidenced by improved Basso–Beattie–Bresnahan locomotor scores compared to untreated injury controls, while concurrently reducing spinal cord water content, indicating effective mitigation of post-traumatic edema formation [234]. The therapeutic benefits of allicin were mechanistically attributed to its potent antioxidant properties, demonstrated through significant reduction of reactive oxygen species levels and enhancement of nicotinamide adenine dinucleotide concentrations, coupled with dose-dependent increases in critical antioxidant enzyme activities, including catalase and superoxide dismutase [234]. Anti-inflammatory effects were evidenced by substantial reductions in nuclear factor-κB and tumor necrosis factor-α levels, key mediators of neuroinflammatory responses that contribute to secondary injury progression [234]. The molecular basis for allicin’s neuroprotective action involves coordinated regulation of the HSP70/Akt/iNOS signaling network, wherein allicin upregulated heat shock protein 70 expression and mRNA levels to enhance cellular stress responses, promoted Akt phosphorylation and PI3K expression to facilitate neuronal survival and regenerative processes, and downregulated inducible nitric oxide synthase protein expression to limit excessive nitric oxide-mediated cytotoxicity and apoptosis [234]. These findings suggest that allicin is a promising therapeutic compound for traumatic spinal cord injury management, offering a multi-target approach that addresses the complex pathophysiology of spinal cord trauma through coordinated antioxidant, anti-inflammatory, and cytoprotective mechanisms that collectively promote tissue preservation and functional recovery [234].

Allicin exhibits potent neuroprotective properties against glutamate-induced excitotoxicity in primary spinal cord neurons [235]. Experimental evaluation demonstrated that allicin treatment effectively mitigated multiple indicators of glutamate-mediated neuronal injury, including significant reductions in lactate dehydrogenase release, enhanced cell viability preservation, and decreased apoptotic neuronal death, establishing its cytoprotective efficacy against excitotoxic insults [235]. The neuroprotective mechanism was fundamentally attributed to allicin’s capacity to attenuate oxidative stress through comprehensive antioxidant effects, evidenced by reduced reactive oxygen species generation, diminished lipid peroxidation, and preservation of endogenous antioxidant enzyme activities that collectively maintain cellular redox homeostasis under pathological conditions [235]. Western blot analysis revealed that allicin’s protective effects were mediated through selective downregulation of inducible nitric oxide synthase expression following glutamate exposure, while neuronal nitric oxide synthase remained unaffected, indicating targeted modulation of pathological nitric oxide production without disrupting physiological nitric oxide signaling [235]. Simultaneously, allicin significantly upregulated heat shock protein 70 expression at both transcriptional and translational levels, establishing this molecular chaperone as a critical component of the neuroprotective response [235]. The essential role of HSP70 in allicin’s therapeutic mechanism was definitively established through RNA interference studies, where HSP70 knockdown not only attenuated allicin’s neuroprotective capacity but also partially abolished its regulatory effects on iNOS expression, demonstrating the interdependent nature of these molecular pathways [235]. These findings collectively establish allicin as a promising therapeutic candidate for spinal cord injury treatment, operating through a precisely defined HSP70/iNOS regulatory mechanism that coordinates cellular stress responses and oxidative damage mitigation to promote neuronal survival and tissue preservation in the context of excitotoxic injury [235].

### 3.3. Asiatic Acid

Asiatic acid and its derivative—asiaticoside—present a noteworthy therapeutic avenue for spinal cord injury management via diverse mechanisms [236,237]. Sourced from Centella asiatica, these compounds are recognized for their anti-inflammatory and antioxidant attributes, which play a pivotal role in alleviating secondary damage cascades post-SCI [236,237].

Asiatic acid demonstrates significant neuroprotective efficacy in traumatic spinal cord injury through coordinated attenuation of oxidative stress, inflammatory responses, and enhancement of functional recovery outcomes [237]. Experimental evaluation in rat models revealed that immediate post-injury administration of asiatic acid substantially reduced lipid peroxidation damage, as evidenced by significantly decreased malondialdehyde levels (2.622 versus 5.525 in trauma controls, *p* = 0.001), indicating effective mitigation of oxidative cellular damage that characterizes secondary injury cascades [237]. The anti-inflammatory properties of asiatic acid were demonstrated through significant reductions in key pro-inflammatory cytokines, including tumor necrosis factor-α (32.036 versus 55.182 in controls, *p* = 0.026) and interleukin-1β (86.685 versus 123.02 in controls, *p* = 0.016), while interleukin-6 levels showed a trend toward reduction that did not reach statistical significance (155.0 versus 215.5, *p* = 0.219) [237]. Most importantly, these molecular improvements translated into meaningful functional benefits, with asiatic acid-treated animals achieving significantly superior Tarlov motor recovery scores (3.00 versus 1.375 in trauma controls, *p* = 0.001), demonstrating enhanced locomotor function restoration following spinal cord trauma [237]. Control studies confirmed that the polyethylene glycol vehicle did not contribute to the observed therapeutic effects, with no significant differences between vehicle and trauma groups across functional and inflammatory parameters, while the significant difference in malondialdehyde levels between trauma and vehicle groups (*p* = 0.002) validated the oxidative stress model [237]. These comprehensive findings show asiatic acid as a promising therapeutic intervention for acute spinal cord injury management, offering a multi-target approach that addresses the complex pathophysiology of spinal cord trauma through coordinated antioxidant and anti-inflammatory mechanisms that collectively promote tissue preservation and functional recovery in the critical post-injury period [237].

Asiaticoside demonstrates comprehensive neuroprotective efficacy in spinal cord injury through multifaceted mechanisms that target both cellular pathology and functional recovery outcomes [236]. Functional assessment revealed that asiaticoside treatment significantly accelerated the restoration of critical physiological functions, including markedly reduced time to spontaneous urination recovery and enhanced motor function restoration, as demonstrated through multiple validated assessment methods including Basso–Beattie–Bresnahan scoring, inclined plate grasp experiments, and footprint analysis [236]. The molecular basis for these functional improvements was evidenced by asiaticoside’s capacity to modulate key pathophysiological processes, including upregulation of neuritin levels in spinal cord tissue, which supports neuronal growth and regeneration, coupled with a significant reduction of tumor necrosis factor-alpha concentrations, which indicates effective anti-inflammatory activity [236]. Critically, asiaticoside treatment substantially attenuated apoptotic cascades, as demonstrated by reduced caspase-3 levels, a key executioner enzyme in programmed cell death pathways, while Fluoro-Jade B staining provided direct confirmation of decreased neuronal apoptosis in treated animals [236]. Morphological analysis revealed superior preservation of neuronal structural integrity in asiaticoside-treated groups compared to untreated controls, indicating robust cytoprotective effects that maintain cellular architecture essential for functional recovery [236].

### 3.4. Curcumin

Curcumin, a polyphenolic compound derived from Curcuma longa rhizomes, presents a promising therapeutic avenue for spinal cord injury treatment, owing to its inherent anti-inflammatory and antioxidant properties [238,239,240,241]. Investigations suggest that curcumin is capable of modulating multiple pathophysiological pathways implicated in SCI, thereby attenuating inflammation and oxidative stress while fostering functional recovery [238,239,240,242].

Razavi et al. proved that curcumin demonstrates significant therapeutic potential in spinal cord injury management through its capacity to modulate critical signaling networks that drive secondary injury cascades and limit recovery outcomes [240]. The compound exerts its neuroprotective effects by strategically targeting three fundamental cellular pathways: nuclear factor erythroid 2-related factor 2 (Nrf2), nuclear factor kappa B (NF-κB), and transforming growth factor beta (TGF-β), each of which plays a pivotal role in determining the extent of tissue damage and functional impairment following spinal cord trauma [239]. Through modulation of the Nrf2 pathway, curcumin enhances endogenous antioxidant responses that counteract oxidative stress-mediated cellular damage, while its effects on NF-κB signaling attenuate pro-inflammatory cascades that contribute to secondary injury progression and tissue destruction [239]. Additionally, curcumin’s influence on TGF-β pathways addresses fibrotic responses and glial scar formation that impede axonal regeneration and functional recovery [239]. The coordinated modulation of these interconnected signaling networks enables curcumin to address multiple pathological processes simultaneously, effectively attenuating the severity of spinal cord injury complications through a comprehensive therapeutic approach [239].

Curcumin demonstrates comprehensive neuroprotective efficacy in spinal cord injury through autophagy-mediated mechanisms that address multiple pathological processes and promote functional recovery while concurrently reducing neuronal apoptosis, indicating robust cytoprotective effects that preserve cellular viability in the post-injury environment [243]. The therapeutic benefits extended to structural improvements, with curcumin treatment promoting spinal cord tissue integrity restoration and enhanced remyelination processes that are critical for axonal function and signal transmission recovery [243]. Anti-inflammatory properties were demonstrated through effective suppression of inflammatory responses that typically exacerbate secondary injury cascades and impede recovery potential [243]. Mechanistic analysis revealed that curcumin’s neuroprotective effects were fundamentally mediated through enhancement of autophagy, a cellular quality control mechanism that removes damaged organelles and proteins to maintain cellular homeostasis, coupled with inhibition of the Akt/mTOR signaling pathway, a key regulator of cellular metabolism and autophagy activation [243]. The critical role of autophagy in curcumin’s therapeutic mechanism was validated through functional studies demonstrating that autophagy inhibition partially eliminated curcumin’s protective effects on spinal cord injury outcomes, confirming the dependence of therapeutic efficacy on this cellular process [243].

Curcumin’s primary therapeutic mechanism involves activation of the nuclear factor erythroid-2-related factor 2/heme oxygenase 1 (Nrf2/HO-1) signaling pathway, a critical cellular defense system that regulates antioxidant responses and cytoprotective gene expression to counteract oxidative stress-mediated tissue damage characteristic of spinal cord injury pathophysiology [244]. Its potent free radical scavenging capacity directly addresses the deleterious effects of oxygen-derived free radicals and high-energy oxidants that serve as primary mediators of secondary spinal cord injury, providing essential antioxidant protection that limits cellular damage and tissue destruction in the post-injury environment [244]. The compound’s anti-inflammatory properties are mediated through inhibition of nuclear factor-κ-light-chain-enhancer of activated B cells (NF-κB), a key transcriptional regulator of inflammatory gene expression, thereby curtailing inflammatory damage that contributes to secondary injury cascades and impedes recovery potential [244]. The convergence of these complementary mechanisms—Nrf2/HO-1 pathway activation for enhanced cellular defense, direct free radical scavenging for oxidative stress mitigation, and NF-κB inhibition for inflammatory suppression—positions curcumin as an attractive therapeutic candidate with a minimal adverse effects profile [244].

Yuan et al. found that curcumin demonstrates significant therapeutic potential for spinal cord injury recovery through targeted inhibition of critical signaling pathways that regulate inflammatory responses and glial scar formation, two fundamental processes that determine injury severity and regenerative capacity [245]. The compound exerts its therapeutic effects by inhibiting nuclear factor kappa-light-chain-enhancer of activated B cells (NF-κB) expression and suppressing the transforming growth factor beta (TGF-β)-SRY-related high mobility group box gene 9 (SOX9) signaling pathway, creating a coordinated intervention that addresses multiple pathological processes simultaneously [245]. The mechanistic relationship between these pathways is critical, as NF-κB activation typically leads to upregulation of TGF-β-SOX9 signaling, which subsequently promotes glial scar formation through TGF-β-mediated cellular responses that create physical and molecular barriers to axonal regeneration [245]. By inhibiting both NF-κB and SOX9 signaling cascades, curcumin effectively disrupts this pathological sequence, reducing inflammatory damage while simultaneously limiting glial scar formation that impedes neuronal regeneration and functional recovery [245]. The dual inhibition of these interconnected pathways positions curcumin as a promising therapeutic intervention with significant potential for promoting neuronal regeneration following spinal cord injury, offering a mechanistically sound approach to addressing the complex pathophysiology that underlies poor recovery outcomes and establishing a foundation for enhanced restoration of neuronal function after traumatic spinal cord injury [245].

In their study, Bang et al. found that curcumin exhibits optimal neurogenic effects at 1 µmol/L concentration, promoting spinal cord neural stem/progenitor cell (SC-NSPC) proliferation through a biphasic dose–response relationship, while higher concentrations (≥5 µmol/L) paradoxically decrease proliferative activity, establishing a critical therapeutic window for optimal neuroregeneration [246]. In vivo evaluation revealed that curcumin treatment significantly enhanced SC-NSPC expression following spinal cord injury, as evidenced by increased nestin/BrdU co-immunoreactivity one week post-injury compared to vehicle controls, indicating robust stimulation of endogenous neural stem cell populations that are essential for tissue repair and functional recovery [246]. The compound’s anti-fibrotic properties were demonstrated through marked reduction in glial fibrillary acidic protein immunoreactivity four weeks post-injury, indicating significant attenuation of reactive astrogliosis and glial scar formation that typically impede axonal regeneration and limit recovery potential [246]. Structural improvements were further validated by a significant reduction in lesion cavity volume six weeks post-injury, coupled with observable neurogenesis in peri-lesional areas, demonstrating curcumin’s capacity to promote tissue preservation and cellular regeneration [246]. These molecular and structural benefits translated into meaningful functional improvements, with curcumin-treated animals achieving significantly superior Basso–Beattie–Bresnahan scores compared to controls, with enhanced motor function maintained throughout the six-week evaluation period [246].

In vitro studies revealed that curcumin exhibits dose-dependent cytoprotective effects against tumor necrosis factor-α-induced apoptosis in hUC-MSCs, with higher concentrations providing greater cellular survival rates, as confirmed through lactate dehydrogenase release analysis and flow cytometry assessments [247]. The molecular mechanism underlying curcumin’s protective effects involves selective activation of the ERK1/2 signaling pathway, as evidenced by specific enhancement of ERK1/2 phosphorylation in apoptotic cells, while JNK and P38 pathways remained unaffected, indicating targeted modulation of pro-survival signaling cascades [247]. The specificity of this mechanism was validated through pharmacological intervention studies demonstrating that U0126, an ERK1/2 antagonist, completely reversed curcumin’s effects on ERK1/2 phosphorylation, confirming the pathway-dependent nature of the therapeutic response [247]. Translation to in vivo spinal cord injury models revealed that combined curcumin and hUC-MSC transplantation therapy produced significant improvements in motor function scores at eight weeks post-injury, accompanied by markedly increased survival of transplanted HNA-positive cells within the injured spinal cord tissue [247]. The critical role of ERK1/2 signaling in therapeutic efficacy was further substantiated by the observation that U0126 administration substantially attenuated both functional recovery and transplanted cell survival, establishing the ERK1/2 pathway as essential for curcumin’s neuroprotective effects [247]. These findings establish curcumin as a promising adjuvant therapy for stem cell-based spinal cord injury treatment, offering enhanced transplanted cell survival and improved functional outcomes through targeted ERK1/2 pathway activation that promotes cellular resilience and therapeutic efficacy in the challenging post-injury microenvironment.

Intraperitoneal curcumin administration effectively attenuated the inflammatory cascade characteristic of ischemia–reperfusion injury, significantly reducing inflammatory cytokine expression that drives secondary tissue damage and impedes recovery processes [248]. The compound’s robust antioxidant properties were evidenced by a substantial reduction in oxidative stress and lipid peroxidation, critical pathological processes that contribute to cellular membrane damage and tissue destruction during reperfusion phases [248]. Curcumin’s cytoprotective effects extended to the prevention of apoptotic cell death, a major contributor to neuronal loss following ischemic insults, while simultaneously enhancing endogenous antioxidant defense mechanisms that provide sustained protection against oxidative damage [248]. Histopathological and ultrastructural analyses revealed that curcumin treatment produced significantly superior tissue preservation compared to both methylprednisolone and saline controls, demonstrating reduced cellular damage and improved tissue architecture maintenance [248]. Most importantly, these molecular and structural improvements translated into meaningful functional benefits, with curcumin-treated animals exhibiting notable enhancement in locomotor function that reflects improved neurological outcomes [248].

Curcumin’s antioxidant properties were evidenced by substantial decreases in nitric oxide levels following spinal cord trauma, addressing a critical pathological mediator that contributes to secondary injury progression and neuronal damage [249]. Additionally, curcumin treatment effectively reduced hydroxyl radical formation and lipid peroxidation, two key oxidative processes that compromise cellular membrane integrity and promote tissue destruction in the post-injury environment [249]. These molecular benefits translated into meaningful therapeutic outcomes, with curcumin-treated animals demonstrating significantly superior motor function recovery compared to controls, indicating enhanced preservation of neurological pathways essential for locomotor activity [249]. Structural analysis further supported curcumin’s neuroprotective effects, revealing significantly increased preservation of spinal cord tissue architecture, suggesting reduced secondary tissue loss and improved maintenance of neural connectivity [249].

Curcumin exerts its neuroprotective effects in spinal cord injury through a sophisticated epigenetic mechanism involving the miR-137-3p/NeuroD1 regulatory axis that modulates microglial inflammatory responses and oxidative stress pathways [250]. In lipopolysaccharide-challenged mouse microglial BV2 cells, curcumin treatment significantly attenuated inflammatory marker expression, including tumor necrosis factor-alpha, interleukin-1 beta, and inducible nitric oxide synthase, demonstrating potent anti-inflammatory and antioxidant properties in an established in vitro spinal cord injury model [250]. The molecular mechanism underlying curcumin’s protective effects involves coordinated regulation of miR-137-3p and NeuroD1 expression, wherein curcumin treatment effectively reversed lipopolysaccharide-induced miR-137-3p downregulation while simultaneously suppressing the upregulation of NeuroD1 protein levels that occurs under inflammatory conditions [250]. Functional validation studies confirmed the essential role of miR-137-3p in curcumin’s neuroprotective mechanism, as anti-miR-137-3p transfection completely abolished curcumin’s protective effects, resulting in elevated inflammatory marker expression despite curcumin treatment, establishing miR-137-3p upregulation as a prerequisite for therapeutic efficacy [250]. Conversely, ectopic NeuroD1 overexpression suppressed curcumin’s protective effects and increased inflammatory mediator levels in treated cells, while NeuroD1 silencing reversed the pro-inflammatory effects of miR-137-3p downregulation, confirming NeuroD1 as a critical downstream target in this regulatory pathway [250]. The regulatory relationship was definitively established through demonstration that miR-137-3p directly targets NeuroD1 expression by binding to its 3’ untranslated region, creating an inverse regulatory relationship that is fundamental to curcumin’s anti-inflammatory and antioxidant effects [250].

Curcumin demonstrates superior neuroprotective efficacy compared to methylprednisolone sodium succinate in experimental spinal cord injury through coordinated antioxidant mechanisms that promote functional recovery and tissue preservation [251]. Functional assessment utilizing inclined plane scoring and Basso–Beattie–Bresnahan scale evaluation at 24 h post-trauma revealed that curcumin treatment produced significant neurological outcome improvements, establishing enhanced motor function recovery compared to standard corticosteroid therapy [251]. The molecular basis for these functional improvements was substantiated by comprehensive biochemical analysis demonstrating that curcumin treatment significantly reduced tissue malondialdehyde levels, a key marker of lipid peroxidation and oxidative damage, while concurrently enhancing endogenous antioxidant enzyme activities including glutathione peroxidase, superoxide dismutase, and catalase [251]. This coordinated modulation of oxidative stress markers indicates that curcumin effectively restores cellular redox homeostasis by simultaneously reducing oxidative damage and strengthening antioxidant defense mechanisms that are critical for neuronal survival in the post-injury environment [251]. Light microscopic examination further validated curcumin’s therapeutic efficacy through demonstration of superior tissue structure preservation, indicating reduced secondary tissue damage and improved maintenance of spinal cord architecture compared to controls [251].

Curcumin demonstrates potent anti-neuroinflammatory effects through a sophisticated microRNA-mediated mechanism that targets fundamental inflammatory signaling pathways in activated microglial cells [252]. The compound effectively suppressed lipopolysaccharide-induced inflammatory responses, as evidenced by significant reductions in phosphorylated-p65 levels, a critical marker of nuclear factor-kappa B activation, alongside decreased expression of key pro-inflammatory mediators, including inducible nitric oxide synthase, tumor necrosis factor-alpha, and interleukin-1β [252]. Mechanistic analysis revealed that curcumin’s anti-inflammatory effects are mediated through coordinated modulation of miR-199b-5p and IκB kinase β expression, with curcumin treatment increasing miR-199b-5p levels while simultaneously decreasing IKKβ expression in activated microglial cells [252]. The regulatory relationship between these molecular targets was established through the demonstration that miR-199b-5p directly targets and suppresses IKKβ expression, creating a negative feedback loop that attenuates inflammatory signaling [252]. Functional validation studies confirmed the essential role of this miR-199b-5p/IKKβ axis in curcumin’s therapeutic mechanism, as knockdown of miR-199b-5p or overexpression of IKKβ reversed curcumin’s inhibitory effects on inflammatory responses and NF-κB activation, while IKKβ silencing abolished miR-199b-5p-stimulated inflammatory cytokine production and NF-κB activation [252].

Liu et al. found that curcumin has a distinctive biphasic therapeutic profile in spinal cord injury treatment that reveals superior long-term efficacy compared to standard methylprednisolone therapy despite initial differences in acute-phase responses [253]. During the initial 14-day post-injury period, methylprednisolone exhibited superior therapeutic effects, as evidenced by more substantial improvements in Basso–Beattie–Bresnahan functional scores compared to curcumin-treated animals, with this acute-phase advantage further supported by histological, immunohistochemical, and ultrastructural analyses that confirmed methylprednisolone’s more pronounced early curative effects [253]. However, a critical therapeutic transition occurred after the 14-day timepoint, where curcumin’s effectiveness became markedly more apparent than methylprednisolone’s, indicating a fundamental shift in therapeutic dynamics that favors curcumin’s sustained neuroprotective mechanisms over the acute anti-inflammatory effects of corticosteroid treatment [253]. This temporal pattern suggests that while methylprednisolone provides rapid suppression of acute inflammatory responses that dominate the early injury phase, curcumin’s multifaceted neuroprotective properties—including antioxidant effects, cellular repair mechanisms, and tissue regeneration support—become increasingly advantageous during the chronic recovery phase when tissue healing and functional restoration are paramount [253].

Tetrahydrocurcumin demonstrates comprehensive neuroprotective efficacy in spinal cord injury through multifaceted mechanisms that address inflammation, oxidative stress, apoptosis, and tissue degradation while promoting cellular survival pathways [254]. Functional assessment revealed that tetrahydrocurcumin treatment significantly enhanced Basso–Beattie–Bresnahan motor scores at two weeks post-injury and effectively inhibited spinal cord water accumulation, indicating improved neurological function and reduced edema formation that typically impedes recovery [254]. The compound’s anti-inflammatory properties were evidenced by significant suppression of key inflammatory mediators, including nuclear factor-kappa B p65, tumor necrosis factor-alpha, interleukin-1 beta, and interleukin-6, which are elevated following spinal cord injury and contribute to secondary tissue damage [254]. Tetrahydrocurcumin’s robust antioxidant effects were demonstrated through substantial reduction of malondialdehyde levels coupled with enhanced activity of critical antioxidant enzymes, including superoxide dismutase, glutathione, and glutathione peroxidase, indicating restoration of cellular redox homeostasis and protection against oxidative tissue damage [254]. Anti-apoptotic effects were confirmed through significant suppression of caspase-3 activity and B-cell lymphoma 2-associated X protein expression, key mediators of programmed cell death that contribute to neuronal loss following injury [254]. Additionally, tetrahydrocurcumin effectively inhibited matrix metalloproteinase-3, matrix metalloproteinase-13, and cyclooxygenase-2 expression, enzymes responsible for extracellular matrix degradation and inflammatory processes that exacerbate tissue damage and impede recovery [254]. The therapeutic mechanism involves activation of survival signaling pathways, as evidenced by enhanced phosphorylated Akt and forkhead box O4 protein levels, indicating modulation of PI3K/Akt/mTOR signaling and FOXO4-mediated oxidative stress responses that promote cellular survival and tissue preservation [254].

### 3.5. Edaravone

Edaravone, recognized for its free radical scavenging capabilities, has emerged as a therapeutic agent for spinal cord injuries, primarily due to its capacity to alleviate secondary injury mechanisms and facilitate functional recovery [255,256,257,258]. Its neuroprotective effects are largely attributed to the inhibition of lipid peroxidation and the reduction of oxidative stress, both of which play pivotal roles in the pathophysiology of SCI [255,256,258].

Edaravone’s primary mechanism involves comprehensive regulation of ferroptosis pathways, particularly during the acute injury phase, where edaravone treatment significantly upregulates anti-ferroptosis proteins, including glutathione peroxidase 4 and system Xc-light chain, while simultaneously downregulating pro-ferroptosis mediators such as Acyl-CoA synthetase long-chain family member 4 and 5-lipoxygenase, with these effects being most pronounced within two days post-injury when ferroptotic cell death predominantly occurs [258]. The neuronal specificity of edaravone’s effects is evidenced by targeted modulation of GPX4/ACSL4/5-LOX expression in spinal segments below the lesion site and in motor neurons, indicating that the compound effectively reverses secondary injury progression by inhibiting ferroptosis in regions critical for locomotor function [258]. Anti-inflammatory properties are demonstrated through upregulation of anti-inflammatory cytokines, including interleukin-10, interleukin-13, and adiponectin, coupled with significant reduction in microgliosis and astrogliosis that collectively indicate substantial neuroinflammation attenuation during the acute post-injury phase [258]. These molecular improvements translate into meaningful long-term therapeutic outcomes, with edaravone treatment enhancing neuronal survival, promoting spinal cord tissue sparing, and reducing cavity formation at the injury epicenter, while functional assessments, including BBB scores and Catwalk gait analysis, demonstrate significant improvements in hindlimb movement and coordination that persist from 3 to 8 weeks post-injury [258]. Electrophysiological measures, including enhanced motor-evoked potential amplitude, further corroborate the anatomical and locomotor recovery benefits [258].

Edaravone demonstrates significant therapeutic potential in spinal cord injury recovery through its capacity to enhance bone marrow mesenchymal stem cell differentiation into functional neuron-like cells, resulting in superior functional outcomes and tissue regeneration compared to conventional stem cell therapy alone [257]. Li et al. successfully isolated high-purity bone marrow mesenchymal stem cells with characteristic surface antigen expression profiles (CD44: 95.5%, CD90: 91.7%, CD29: 90.7%) while lacking hematopoietic markers (CD34 and CD45), confirming their nonhematopoietic stem cell identity suitable for neuronal differentiation applications [257]. Edaravone treatment at the optimal concentration of 20 mg/L effectively induced morphological transformation of BMSCs into neuron-like cells, with cells progressing from rounded structures following basic fibroblast growth factor preinduction to polygonal shapes with prominent protuberances, ultimately developing bipolar, multipolar, and conical neuronal morphologies within five hours of edaravone exposure [257]. The neuronal differentiation was biochemically validated through dramatically increased neuron-specific enolase expression (98.6% positive rate versus 68% in positive controls), enhanced expression of neuronal markers including nestin and neurofilament 200, decreased glial fibrillary acidic protein levels, and scanning electron microscopy confirmation of axon-like structure extension [257]. In vivo translation revealed that edaravone-pretreated BMSC transplantation significantly enhanced locomotor function recovery as assessed by BBB scale evaluation, with treated animals demonstrating markedly faster and superior hindlimb functional recovery compared to conventional BMSC transplantation groups [257]. Histological analysis confirmed superior tissue regeneration outcomes, including attenuated spinal cord tissue spacing, increased neuronal populations, reduced necrotic cavity formation, and improved gray and white matter organization that progressed with extended transplantation duration [257]. Ultrastructural examination revealed reduced cytoplasmic edema, attenuated mitochondrial vacuolation, decreased nuclear chromatin concentration, and progressive normalization of mitochondrial and endoplasmic reticulum structures, while nerve fiber arrangement became increasingly standardized with decreased intercellular spacing due to regenerated neuronal filling [257]. Cell survival and neural repair were confirmed through BrdU-positive cell distribution throughout injured areas and enhanced biotin dextran amine tracer fluorescence signals [257].

The combined treatment of edaravone and neural stem cell transplantation demonstrates superior therapeutic efficacy in complete spinal cord transection repair compared to either intervention alone, achieving enhanced functional recovery, improved cell survival, and increased nerve fiber regeneration [259]. Functional assessment using BBB rating scales revealed that all treatment groups (edaravone, neural stem cell transplantation, and combination therapy) achieved significantly better recovery than controls (*p* < 0.01), with the combination group demonstrating the most rapid recovery trajectory and achieving the highest functional scores at 8 weeks post-injury (11.21 ± 0.14) compared to edaravone alone (8.46 ± 0.1), transplantation alone (8.54 ± 0.13), and controls (4.21 ± 0.11) [259]. Notably, only the combination group achieved recovered motion coordination between front and rear limbs with restored palm load-bearing capacity by 8 weeks, while individual treatments showed poor coordination without significant differences between them (*p* > 0.05) [259]. Cell survival analysis through PKH-26 labeling confirmed the superior therapeutic environment created by combination therapy, with the edaravone plus transplantation group exhibiting significantly higher numbers of surviving transplanted neural stem cells (68.62 ± 9.44) compared to transplantation alone (31.58 ± 7.64), indicating that edaravone enhances the survival and integration of transplanted cells within the hostile post-injury microenvironment [259]. The functional benefits were corroborated by nerve fiber regeneration assessment using FG retrograde tracing, which revealed that the combination group achieved the highest numbers of FG-labeled pyramidal cells and axons (42.6 ± 5.1) compared to neural stem cell transplantation alone (29.4 ± 4.0), edaravone alone (24.6 ± 3.6), and controls (9.6 ± 2.1), with the combination group showing statistically significant superiority (*p* < 0.01) and a direct correlation between FG-positive fiber counts and functional BBB scores, indicating restoration of axoplasmic transportation function [259].

### 3.6. Epigallocatechin

Epigallocatechin gallate, a prominent polyphenol in green tea, exhibits neuroprotective properties relevant to spinal cord injuries [260,261]. Research suggests that EGCG’s antioxidative, anti-inflammatory, and anti-edema mechanisms can alleviate secondary damage following SCI [260,261,262]. Zhu et al. observed a decrease in malondialdehyde concentrations in EGCG-treated groups compared to controls, indicating effective suppression of oxidative stress-mediated tissue destruction that characterizes secondary injury cascades following spinal cord trauma [262]. This antioxidant effect is particularly significant given that lipid peroxidation represents a fundamental pathological process that compromises cellular membrane integrity, disrupts cellular function, and contributes to progressive tissue damage in the post-injury environment [262].

Epigallocatechin demonstrates significant anti-edema efficacy in acute spinal cord injury through targeted modulation of the p38MAPK/NF-κB signaling pathway [262]. The compound’s anti-inflammatory properties were evidenced by substantial reductions in tumor necrosis factor-alpha and interleukin-1 beta release following spinal cord injury, with EGCG demonstrating more pronounced TNF-α suppression than the specific p38MAPK inhibitor SB203580 while achieving equivalent IL-1β reduction, indicating potent anti-inflammatory activity that extends beyond single pathway inhibition [262]. Mechanistic analysis revealed that EGCG effectively disrupts the p38MAPK/NF-κB/AQP4 signaling cascade by significantly downregulating phosphorylated p38MAPK protein expression while maintaining total p38MAPK levels unchanged, suggesting selective inhibition of pathway activation rather than general protein suppression [262]. The downstream effects included marked reduction in NF-κB p65 expression, confirming the proportional relationship between phosphorylated p38MAPK and NF-κB p65 activation, while aquaporin-4 protein levels, which were dramatically elevated following spinal cord injury, were significantly reduced by EGCG treatment, indicating targeted modulation of this critical water channel protein involved in spinal cord fluid homeostasis [262]. The molecular pathway inhibition translated into meaningful clinical benefits, with EGCG treatment producing a superior reduction in spinal cord water content compared to SB203580, directly correlating with enhanced anti-edema effects that address one of the most critical pathophysiological processes in secondary spinal cord injury [262].

### 3.7. Estrogen

Estrogen has garnered attention as a potential neuroprotective agent for spinal cord injuries, with research indicating its capacity to reduce damage and enhance recovery [263,264]. These beneficial effects are thought to stem from estrogen’s anti-inflammatory, antioxidant, and anti-apoptotic properties, which collectively aid in the maintenance of neural tissue and function following SCI [263].

Estrogen demonstrates dose-dependent neuroprotective efficacy in acute spinal cord injury through comprehensive modulation of inflammatory and apoptotic pathways that preserve neuronal survival in both lesion and penumbral regions [265]. The experimental evaluation utilized a controlled design comparing varying low-dose estrogen treatments (1, 5, 10, or 100 μg/kg) against vehicle-treated injured rats and laminectomy controls at 48 h post-injury, with sham-operated animals exhibiting minimal pro-inflammatory responses, proteolytic activity, and neuronal death, establishing baseline parameters for comparison [265]. Vehicle-treated spinal cord injury animals displayed the characteristic pathological profile of secondary injury, including significant pro-inflammatory responses, reactive gliosis, elevated expression and activity of proteolytic enzymes including calpain and caspase-3, increased Bax/Bcl-2 ratios indicative of pro-apoptotic signaling, and substantial neuronal death extending from the lesion epicenter to caudal spinal cord regions [265]. Estrogen treatment across all tested doses effectively attenuated these pathological processes, demonstrating significant reduction in pro-inflammatory and proteolytic activities while providing robust neuronal protection in the caudal penumbra, a critical region where secondary injury progression determines functional outcomes [265]. Notably, the dose–response analysis revealed that 10 μg/kg estrogen achieved equivalent therapeutic efficacy to the 100 μg/kg dose across all measured parameters, establishing an optimal therapeutic window that maximizes neuroprotective benefits while minimizing potential dose-related complications [265].

Sribnick et al. showed that estrogen-treated animals exhibited significantly enhanced survival rates and superior locomotor function compared to vehicle controls, with improvements evident as early as 3 days post-injury and persisting throughout the 42-day evaluation period, culminating in average BBB scores of approximately 13 that indicated restored weight-bearing capacity, coordinated stepping patterns, and hindlimb/forelimb coordination that contrasted markedly with the impaired plantar stepping observed in untreated animals [266]. Histological analysis confirmed estrogen’s tissue-protective effects, with treated animals showing significantly reduced percentages of damaged spinal cord tissue in both lesion and penumbral regions, achieving damage levels comparable to sham controls in penumbral areas and demonstrating preserved white matter integrity that underlies functional recovery [266]. The anti-inflammatory properties of estrogen were evidenced through marked reduction in COX-2 enzyme activity, attenuation of NF-κB nuclear translocation with reversal of IκB-α degradation, decreased astroglial reactivity as measured by reduced GFAP immunoreactivity in both gray and white matter, and substantial reduction in activated macrophage and microglial populations throughout the injury site and caudal penumbra [266]. Neuroprotective mechanisms included maintenance of neuronal density comparable to sham animals despite persistent cystic formation, inhibition of apoptotic signaling through attenuation of the Bax/Bcl-2 ratio, significant reduction in calpain and caspase-3 proteolytic activity that contributes to neurodegeneration, and preservation of axonal integrity, as demonstrated by decreased dephosphorylated neurofilament protein levels in lesion areas [266].

### 3.8. Gastrodin

Gastrodin (GAS), a bioactive compound derived from the traditional Chinese herb Gastrodia elata, has shown promising therapeutic potential in the treatment of spinal cord injuries [267]. Its effects are primarily attributed to its antioxidant and anti-inflammatory properties, which contribute to the amelioration of SCI-related symptoms and promote recovery [267]. In experimental models of contusive SCI, GAS administration resulted in marked improvements in locomotor function, as evidenced by enhanced Basso–Beattie–Bresnahan scores from 7 to 28 days post-injury, indicating substantial functional recovery compared to untreated controls [267]. The compound’s neuroprotective effects are mediated through preservation of blood–spinal cord barrier integrity, demonstrated by significantly reduced Evans blue extravasation and decreased vascular permeability [267]. GAS exhibits potent anti-inflammatory properties by suppressing the upregulation of key proinflammatory cytokines, including tumor necrosis factor-alpha (TNFα) and interleukin-1 beta (IL-1β), which are critical mediators of secondary tissue damage following SCI [267]. Additionally, the compound effectively mitigates oxidative stress by preventing lipid peroxidation, as indicated by reduced TBARS content, while simultaneously preserving endogenous antioxidant defenses through maintenance of glutathione levels and superoxide dismutase activity [267]. The molecular basis for these protective effects involves upregulation of the Nrf2-GCLc/GCLm signaling pathway, wherein GAS enhances the expression of nuclear factor (erythroid-derived 2)-like 2 (Nrf2) and its downstream effectors, the catalytic and modified subunits of γ-glutamylcysteine ligase, thereby promoting cellular antioxidant capacity and coordinating the anti-inflammatory response [267].

### 3.9. Ginko Biloba Extract 761

Ginkgo biloba extract 761 (EGb761) has been investigated for its capacity to provide neuroprotection in the context of spinal cord injuries, yielding encouraging outcomes in animal models [268,269,270,271]. The extract shows promise in alleviating secondary injury mechanisms, including apoptosis and inflammation, thereby fostering enhanced functional recovery [269,270,271].

Ginkgo biloba extract 761 demonstrates significant therapeutic efficacy in acute spinal cord injury through complementary functional and histopathological improvements that collectively enhance recovery outcomes [270]. Following experimental SCI in rats, EGb761 treatment resulted in substantial functional recovery, as evidenced by significantly improved Basso, Beattie, and Bresnahan scores at 14 days post-injury compared to saline-treated controls (*p* < 0.05), indicating enhanced locomotor capability [270]. This functional improvement was further substantiated by detailed gait analysis, which revealed that EGb761-treated animals exhibited increased stride length, decreased stride width, and reduced toe dragging at 14 days post-injury, collectively demonstrating superior motor coordination and ambulatory function compared to controls (*p* < 0.05) [270]. At the histological level, EGb761 treatment provided robust neuroprotection by significantly reducing tissue necrosis and cavitation at the injury epicenter, as demonstrated through hematoxylin and eosin staining analysis at 14 days post-injury (*p* < 0.05) [270]. The extract’s cytoprotective effects were particularly evident in its ability to attenuate cellular apoptosis, with TUNEL and caspase-3 staining revealing significantly reduced apoptotic cell populations at both 3 and 7 days post-injury compared to control groups [270]. Notably, the temporal pattern of apoptosis differed between tissue types, with gray matter apoptosis peaking at 24 h post-injury while white matter apoptosis demonstrated a delayed onset, increasing at 3 days and reaching maximum levels at 7 days post-injury [270].

In primary spinal cord neurons isolated from embryonic day 14 rats, hydrogen peroxide (H_2_O_2_) administration significantly reduced neuronal survival, inducing apoptotic cell death, as confirmed by terminal deoxynucleotidyl transferase-mediated dUTP nick-end labeling (TUNEL) and Hoechst 33342 nuclear staining [271]. EGb761 treatment provided robust cytoprotection, significantly reversing H_2_O_2_-induced neuronal death in a dose-dependent manner, indicating a direct therapeutic relationship between extract concentration and neuroprotective efficacy [271]. The primary mechanism underlying this protection involves potent antioxidant activity, as H_2_O_2_ exposure resulted in marked elevation of intracellular free radical generation, which EGb761 almost completely reversed, demonstrating that inhibition of reactive oxygen species production is fundamental to the extract’s anti-apoptotic action [271]. Additionally, EGb761 exerts neuroprotection through strategic modulation of key apoptotic regulatory genes, addressing the molecular imbalance induced by oxidative stress [271]. While H_2_O_2_ treatment for 12 h dramatically reduced expression of the anti-apoptotic gene Bcl-2 by 70% without affecting pro-apoptotic Bax levels, EGb761 intervention significantly restored Bcl-2 expression while simultaneously inhibiting Bax expression by 2.3-fold [271]. This dual regulatory effect creates a favorable cellular environment that promotes survival by enhancing anti-apoptotic signaling while suppressing pro-death pathways [271].

In vitro studies demonstrated that both glutamate and hydrogen peroxide induced significant neuronal death accompanied by increased expression of phosphorylated cPLA2 (p-cPLA2), indicating activation of this key inflammatory enzyme [272]. EGb761 treatment effectively counteracted these neurotoxic effects by significantly reversing the elevation in p-cPLA2 expression and subsequent neuronal death induced by both glutamate and hydrogen peroxide exposure [272]. The functional significance of cPLA2 inhibition was further evidenced by EGb761’s ability to significantly reduce prostaglandin E2 (PGE2) release, a critical downstream inflammatory metabolite of cPLA2 activity, thereby disrupting the pro-inflammatory cascade that contributes to neuronal injury [272]. Comparative analysis using the selective cPLA2 inhibitor arachidonyl trifluromethyl ketone confirmed the therapeutic importance of cPLA2 suppression, as direct pharmacological inhibition improved neuroprotection against glutamate and hydrogen peroxide-induced death while favorably modulating the Bcl-2/Bax ratio to promote cell survival [272]. Notably, EGb761 demonstrated superior protective efficacy compared to direct cPLA2 inhibition, suggesting additional complementary neuroprotective mechanisms beyond phospholipase inhibition [272]. The molecular basis for EGb761’s cPLA2 regulatory effects involves modulation of the extracellular signal-regulated kinase 1/2 (ERK1/2) signaling pathway, which plays a crucial role in controlling cPLA2 phosphorylation and subsequent activation [272].

### 3.10. Ginsenosides

Ginsenosides, which are biologically active constituents of ginseng, have demonstrated neuroprotective capabilities in the context of spinal cord injuries [273]. Their therapeutic potential is attributed to various biological activities, including anti-inflammatory, anti-apoptotic, and antioxidant effects [273].

Ginsenoside Rb1 (GRb1) treatment effectively alleviates spinal cord injury by simultaneously inhibiting neuronal apoptosis and reducing the expression of proinflammatory factors, providing dual protection against both primary and secondary injury mechanisms [274]. The therapeutic efficacy of GRb1 is fundamentally dependent on its ability to facilitate the expression of microRNA-130b-5p (miR-130b-5p) in spinal cord injury models, as this microRNA upregulation is crucial for the compound’s neuroprotective effects and its capacity to attenuate microglial-mediated neuronal damage [274]. The molecular target of miR-130b-5p has been identified as Toll-like receptor 4 (TLR4), a critical inflammatory mediator that miR-130b-5p directly targets to attenuate activated microglia-induced neuronal injury [274]. This targeted interaction results in the strategic inactivation of the TLR4/nuclear factor-κB (NF-κB) signaling pathway, a key inflammatory cascade that promotes secondary tissue damage following spinal cord trauma [274]. Through this miR-130b-5p-mediated mechanism, GRb1 effectively suppresses the activation of inflammatory pathways in microglia, leading to reduced secretion of proinflammatory cytokines and creation of a more favorable microenvironment for neuronal survival and tissue repair [274].

In experimental spinal cord injury models, ginsenoside Rb1 (Rb1) treatment effectively decreased motor neuron loss while simultaneously promoting functional recovery, indicating that the compound addresses both cellular preservation and physiological restoration [275]. The underlying mechanism of Rb1’s neuroprotective action involves targeted inhibition of autophagy in injured neurons, which correlates with suppression of both neuronal apoptosis and autophagic cell death within the spinal cord injury environment [275]. These in vivo findings were substantiated through complementary in vitro studies using PC12 cells as a neuronal injury model, where Rb1 treatment significantly increased cell viability by suppressing apoptosis through inhibition of excessive autophagy [275]. The mechanistic relationship between autophagy inhibition and neuroprotection was further validated by pharmacological intervention studies, wherein rapamycin-induced autophagy stimulation completely abolished Rb1’s anti-apoptotic effects, definitively establishing that autophagy inhibition is essential for the compound’s cytoprotective properties [275]. These findings reveal that Rb1’s therapeutic strategy addresses the delicate balance between autophagy as a cellular maintenance mechanism and its pathological overactivation following spinal cord trauma, where excessive autophagic activity contributes to neuronal death rather than cellular repair [275].

Following experimental spinal cord injury in rats, ginsenoside Rg3 (GRg3) treatment produced substantial improvements in behavioral motor functions beginning one week post-injury, as evidenced by significantly elevated scores across multiple standardized assessments, including the Basso–Beattie–Bresnahan rating (11.8 ± 1.3 vs. 6.3 ± 0.7), inclined plane test (64.7° ± 2.7° vs. 52.7° ± 1.6°), toe spread test (2.5 ± 0.1 vs. 2.1 ± 0.1), and hind foot bar grab test (1.8 ± 0.1 vs. 1.3 ± 0.1), compared to untreated controls [276]. These functional improvements were accompanied by notable qualitative enhancements, including occasional weight-supported plantar steps, frequent forelimb–hindlimb coordination, improved toe spreading, and enhanced hind foot grasping ability [276]. At the tissue level, GRg3 provided robust histoprotection against the severe damage typically observed following spinal cord injury, including cystic necrosis, hemorrhage, and vacuolation, while significantly reducing lesion area size and restoring motor neuron populations in both rostral and caudal spinal segments [276]. The compound’s neuroprotective effects extend to molecular regulation of apoptotic pathways, as GRg3 treatment significantly reduced pro-apoptotic Bax expression while increasing the protective Bcl-2/Bax ratio, resulting in substantial decreases in TUNEL-positive apoptotic cells in ventral horn gray matter (reduced to ~37% with 30 mg/kg treatment) and Bax-expressing cells (reduced to ~46%) [276]. Furthermore, GRg3 demonstrated potent anti-inflammatory properties by significantly attenuating the upregulation of key pro-inflammatory cytokines, including TNF-α, IL-1β, and IL-6 mRNA expression while simultaneously reducing elevated levels of inducible nitric oxide synthase and cyclooxygenase-2 [276]. The therapeutic benefits of GRg3 are further enhanced by its ability to suppress microglial activation, as evidenced by significant reductions in Iba1 expression, decreased numbers of activated microglia, and reduced microglial cell body size, particularly in perilesional segments where secondary inflammation is most pronounced [276].

Administration of ginsenoside Rd (GS Rd) at doses of 25 and 50 mg/kg significantly improved locomotor function in rats following experimental spinal cord injury, achieving therapeutic outcomes equivalent to those observed with dexamethasone, a standard positive control treatment [277]. The compound’s neuroprotective effects extend beyond functional recovery to encompass direct tissue preservation, as evidenced by reduced spinal cord tissue injury and enhanced neuronal survival within the lesion area [277]. At the molecular level, GS Rd effectively counteracts oxidative stress through strategic modulation of redox homeostasis, significantly decreasing malondialdehyde levels while simultaneously increasing glutathione content and enhancing superoxide dismutase activity, thereby restoring the cellular antioxidant-oxidant balance that is critically disrupted following spinal cord trauma [277]. The anti-inflammatory properties of GS Rd are demonstrated through potent suppression of pro-inflammatory cytokine production, including tumor necrosis factor-α, interleukin-1β, and interleukin-1, effectively attenuating the neuroinflammatory cascade that contributes to secondary tissue damage [277]. Furthermore, GS Rd provides robust cytoprotection by preventing neuronal apoptosis in spinal cord tissue, directly contributing to the observed enhancement in neuronal survival and functional preservation [277]. The molecular basis for these diverse protective effects involves strategic inhibition of the mitogen-activated protein kinase (MAPK) signaling pathway, a critical cellular cascade that is pathologically activated following spinal cord injury and contributes to inflammation, oxidative stress, and apoptotic cell death [277].

### 3.11. Glutathione

Glutathione exerts a notable influence in the context of spinal cord injuries, primarily attributed to its antioxidant capabilities, which facilitate the reduction of oxidative stress and inflammation, thereby fostering recovery [36,194,278]. Following experimental spinal cord injury in rats, a comprehensive analysis of oxidative stress parameters revealed significant disruption of the glutathione antioxidant system, as evidenced by marked decreases in glutathione content accompanied by substantial increases in nitric oxide production [36]. Changes were consistently observed in both whole spinal cord tissue homogenates and isolated mitochondrial fractions, indicating that oxidative stress affects both cellular and subcellular compartments [36].

The study by Wyss et al., involving 20 healthy controls (median age 50 years, 18 men) and 18 traumatic spinal cord injury subjects (median age 50 years, 16 men), revealed significant metabolic alterations in the central nervous system following spinal cord trauma and identified a novel biomarker for rehabilitation potential [278]. Proton magnetic resonance spectroscopy analysis demonstrated distinct metabolic profiles associated with injury severity, as individuals with complete spinal cord injury exhibited increased total N-acetylaspartate and combined glutamate–glutamine levels, while subjects with incomplete paraplegic injury showed reduced total creatine concentrations [278]. Most importantly, the study established a significant correlation between baseline pontine glutathione levels measured approximately 10 weeks post-injury and subsequent improvements in motor scores during rehabilitation in individuals with incomplete subacute spinal cord injury [278]. This finding represents a significant advance in understanding the relationship between remote neurochemical changes and functional recovery potential, as glutathione concentrations in the pons—a brainstem region anatomically distant from typical spinal injury sites—demonstrated predictive value for rehabilitation outcomes [278]. The identification of pontine glutathione as a biomarker suggests that antioxidant capacity in critical brainstem regions may reflect the nervous system’s overall resilience and adaptive potential following spinal cord trauma [278]. These findings have important clinical implications, as non-invasive magnetic resonance spectroscopy assessment of pontine glutathione levels could potentially be integrated into rehabilitation planning to identify patients with greater recovery potential and optimize therapeutic interventions [278].

The therapeutic efficacy of glutathione in spinal cord injury treatment demonstrates remarkable chirality-dependent differences, with d-chiral glutathione (D-GSH) exhibiting superior neuroprotective and regenerative properties compared to its l-chiral counterpart (L-GSH) through enhanced anti-inflammatory mechanisms and cellular interactions [279]. The superior therapeutic efficacy of D-GSH is most prominently demonstrated through its remarkable ability to promote axon regeneration, with treated rats showing statistically significant increases in axon regrowth compared to L-GSH treatment groups (*p* < 0.001), accompanied by significant improvements in secondary damage limitation and motor function recovery (*p* < 0.01) [279]. The molecular basis for D-GSH’s enhanced therapeutic activity involves superior anti-inflammatory mechanisms, as evidenced by its significantly greater capacity to reduce pro-inflammatory cytokines and glial fibrillary acidic protein (GFAP) levels compared to L-GSH (*p* < 0.001), effects that are mediated through strategic inhibition of the mitogen-activated protein kinase (MAPK) signaling pathway [279]. Furthermore, cellular interaction studies using primary cultured macrophages revealed that D-GSH exhibits significantly greater intracellular interaction with activated macrophages compared to L-GSH (*p* < 0.001), providing a mechanistic explanation for its enhanced anti-inflammatory action through more effective modulation of immune cell responses [279]. These findings represent a paradigm shift in understanding glutathione’s therapeutic potential, revealing that chirality-specific molecular interactions can dramatically influence biological activity and therapeutic outcomes [279]. The discovery that D-GSH possesses superior regenerative capabilities positions chiral glutathione as a promising therapeutic intervention not only for spinal cord injury but potentially for other neurological and inflammatory conditions where precise molecular targeting is essential [279].

Baseline assessment of antioxidant status demonstrated that glutathione levels were significantly decreased in older sham mice (14 months old) compared to younger controls (4 months old), establishing that aging inherently compromises the spinal cord’s primary antioxidant defense system [194]. Following spinal cord injury, young mice (4 months old) exhibited the expected spinal cord injury-dependent depletion of glutathione by three days post-injury, indicating a typical stress response that further compromises antioxidant capacity during the critical secondary injury period [194]. Interestingly, the abundance of proteins responsible for glutathione synthesis and recycling remained unaffected by either age or injury, suggesting that the observed glutathione deficits result from increased consumption rather than impaired production capacity [194]. The differential oxidative responses between age groups were further illuminated by glutathione peroxidase activity patterns, as young mice showed increased enzyme activity only after spinal cord injury, while older mice demonstrated constitutively elevated glutathione peroxidase activity even in sham conditions, indicating chronic oxidative stress in aged spinal cords [194]. This age-related oxidative burden was confirmed by protein oxidation analysis, which revealed that older sham mice had significantly more oxidized proteins (3-nitrotyrosine) in their spinal cords compared to younger controls, with only young mice showing significant injury-induced increases in protein oxidation at three days post-injury [194]. The clinical implications of these age-related differences became apparent through therapeutic intervention studies using N-acetylcysteine-amide (NACA), which successfully restored glutathione levels and improved the redox environment in both age groups at one day post-injury, demonstrating acute biochemical efficacy [194]. However, prolonged NACA treatment revealed striking age-dependent therapeutic disparities, as three days of treatment failed to improve motor, sensory, or anatomical outcomes in young mice at 28 days post-injury while paradoxically trending toward toxicity across all measured outcomes in older mice [194]. These findings establish that aging fundamentally alters spinal cord oxidative homeostasis and therapeutic responsiveness, creating a complex clinical scenario where older individuals present with compromised baseline antioxidant capacity yet demonstrate increased vulnerability to antioxidant interventions, necessitating age-specific treatment protocols that account for differential oxidative stress patterns and therapeutic tolerability in spinal cord injury management [194].

### 3.12. Ligustilide

Ligustilide is a naturally occurring compound found in traditional Chinese medicine [280,281]. It demonstrates significant therapeutic potential in spinal cord injury recovery through dual mechanisms of functional restoration and neuroprotection [281]. In experimental models, ligustilide treatment markedly enhanced motor function recovery, as evidenced by significantly improved Basso–Beattie–Bresnahan scale scores in rats with spinal cord injury, indicating restored locomotor capabilities and enhanced coordination following neural trauma [281]. Beyond functional improvements, ligustilide exerts potent anti-inflammatory and antioxidative effects at the injury site, significantly suppressing the production of key inflammatory mediators including intracellular reactive oxygen species (iROS), prostaglandin E2 (PGE2), interleukin-1β (IL-1β), and tumor necrosis factor-α (TNF-α) [281]. Additionally, the compound effectively downregulates inducible nitric oxide synthase (iNOS) gene expression, further contributing to the reduction of secondary injury cascades that typically exacerbate spinal cord damage [281].

### 3.13. Lycopene

Lycopene, a naturally occurring carotenoid, has shown promising potential in the treatment of spinal cord injuries (SCI) due to its antioxidant and anti-inflammatory properties [282]. The compound demonstrates profound neuroprotective effects in spinal cord injury through comprehensive restoration of blood–spinal cord barrier integrity and enhancement of functional recovery [282]. In experimental mouse models, lycopene treatment significantly improved motor function outcomes compared to untreated controls, with beneficial effects becoming apparent by day 2 post-injury and sustaining through 15 days of observation [282]. The therapeutic mechanisms underlying these improvements involve multifaceted BSCB stabilization, including dramatic reduction of spinal cord edema through decreased tissue water content and marked attenuation of barrier permeability, as demonstrated by reduced Evans blue extravasation into injured tissue [282]. Lycopene’s protective effects are mediated through potent anti-inflammatory actions, specifically suppressing the upregulation of pro-inflammatory cytokines TNF-α and NF-κB that typically contribute to secondary injury cascades following spinal trauma [282]. Critically, lycopene treatment preserved BSCB structural integrity by upregulating essential tight junction proteins zonula occludens-1 (ZO-1) and claudin-5, which are fundamental components of the barrier’s selective permeability function and are typically disrupted following spinal cord injury [282]. Additionally, the compound’s antioxidative properties were evidenced by decreased heme oxygenase-1 (HO-1) expression, indicating reduced oxidative stress burden at the injury site [282].

Lycopene’s potent antioxidative properties were evidenced by significant reduction of oxaliplatin-induced lipid peroxidation in brain tissue, coupled with robust enhancement of endogenous antioxidant defense systems through increased activities of superoxide dismutase (SOD), catalase (CAT), and glutathione peroxidase (GPx), as well as elevated glutathione (GSH) levels [283]. Its anti-inflammatory actions were demonstrated through marked suppression of key inflammatory mediators, including mitogen-activated protein kinase-14 (MAPK14), nuclear factor kappa-B (NF-κB), and tumor necrosis factor-α (TNF-α) in both central and peripheral nervous tissues [283]. Notably, lycopene effectively mitigated oxaliplatin-induced endoplasmic reticulum stress by downregulating critical stress response proteins, including activating transcription factor-6 (ATF6), glucose-regulated protein-78 (GRP78), pancreatic endoplasmic reticulum kinase (PERK), and inositol-requiring enzyme-1 (IRE1) [283]. The neuroprotective effects extended to tissue preservation and neuronal survival, as evidenced by increased neural cell adhesion molecule (NCAM) expression, decreased glial fibrillary acidic protein (GFAP) levels indicating reduced glial activation, and significantly diminished caspase-3 immunopositivity reflecting reduced apoptotic cell death [283]. Furthermore, lycopene enhanced neuroplasticity and repair mechanisms through upregulation of brain-derived neurotrophic factor (BDNF) in sciatic tissue, which is crucial for neuronal survival and regeneration [283].

### 3.14. Melatonin

Melatonin, an indoleamine hormone, has demonstrated significant neuroprotective effects in the context of spinal cord injury, primarily by mitigating secondary injury mechanisms such as inflammation and oxidative stress [284,285,286,287,288]. Its ability to cross the blood–brain barrier contributes to its effectiveness in the central nervous system [288].

Melatonin’s antioxidative properties are associated with its capacity to scavenge free radicals and modulate inflammatory responses, which support neuroprotection and functional recovery [287]. In organotypic spinal cord slice culture models, melatonin administration markedly increased SOD immunopositivity, indicating enhanced cellular capacity to neutralize superoxide radicals that are generated in excessive quantities following spinal trauma and contribute substantially to secondary injury cascades [287]. This enhancement of SOD expression represents a critical therapeutic mechanism, as superoxide dismutase serves as the primary enzymatic defense against superoxide anion-mediated oxidative damage, which is a major contributor to progressive tissue destruction, neuronal death, and functional deterioration in the acute and subacute phases following spinal cord injury [287]. The observed positive influence of melatonin on antioxidative processes suggests that this naturally occurring neurohormone can effectively bolster the injured spinal cord’s intrinsic capacity to combat oxidative stress, potentially limiting the extent of secondary damage that typically expands the initial injury zone and compromises recovery potential [287].

Naseem et al. found that melatonin’s potent anti-inflammatory properties are demonstrated through effective suppression of NF-κβ-regulated adhesion molecules and marked reduction in pro-inflammatory cytokine production, thereby preventing the cascading neuronal damage typically associated with post-traumatic inflammatory responses in neurological disorders [288]. Experimental validation in animal models has consistently demonstrated melatonin’s efficacy in attenuating both inflammation and tissue injury following spinal cord trauma [288]. Critical to melatonin’s therapeutic profile is its regulation of mitogen-activated protein kinase (MAPK) signaling pathways, specifically reducing the activation of p38, JNK, and ERK1/2 kinases, which are pivotal in cellular stress responses and gene expression alterations following spinal cord injury, with studies documenting significant MAPK suppression at 24 h post-trauma using therapeutic doses of 50 mg/kg [288]. Melatonin’s antioxidative mechanisms operate through dual pathways: direct free radical scavenging by both melatonin and its metabolic derivatives and enhancement of endogenous antioxidant enzyme activities, collectively providing robust protection against oxidative stress-mediated secondary injury [288]. The compound’s antioxidative efficacy is further evidenced by its ability to significantly reduce malondialdehyde (MDA) levels—a key marker of lipid peroxidation—while simultaneously restoring depleted glutathione (GSH) concentrations in injured spinal cord tissue [288].

In organotypic spinal cord culture systems, melatonin treatment significantly reduced cellular death while concurrently enhancing overall tissue vitality, with the most pronounced protective effects observed when melatonin was administered prophylactically prior to hydrogen peroxide exposure, indicating its potential value as a preventive therapeutic intervention in anticipated oxidative injury scenarios [286]. Its antioxidative mechanisms were evidenced by its ability to effectively mitigate the depletion of total thiols, which serve as critical indicators of cellular oxidative stress burden and represent essential components of the endogenous antioxidant defense system [286]. Beyond general cytoprotection, melatonin specifically preserved neuronal structural and functional integrity by attenuating the reduction of key neuronal markers, including NeuN (neuronal specific nuclear protein), which is fundamental for neuronal identity and survival, and synaptophysin, a crucial presynaptic protein essential for synaptic vesicle function and neurotransmitter release [286]. The preservation of these neuronal markers suggests that melatonin not only prevents cell death but also maintains the specialized cellular machinery necessary for neuronal communication and synaptic transmission, which are critical for functional recovery following spinal cord injury [286].

Clinical assessment using the Basso Mouse Scale revealed that melatonin treatment significantly enhanced motor function recovery following spinal cord injury, with therapeutic benefits sustained for up to four weeks post-trauma [289]. Histological analysis confirmed melatonin’s neuroprotective capacity, demonstrating significantly increased motor neuron survival in the anterior horn compared to vehicle-treated controls, coupled with a marked reduction in apoptotic cell death that typically contributes to progressive tissue loss after spinal cord injury [289]. Ultrastructural examination via transmission electron microscopy revealed that melatonin effectively preserved mitochondrial integrity by reducing vacuolization and restoring cristae structure that are otherwise severely compromised following spinal trauma [289]. The compound’s antioxidative mechanisms were validated through enhanced superoxide dismutase content and glutathione peroxidase (GSH-PX) activity while simultaneously reducing malondialdehyde (MDA) levels and hydrogen peroxide-induced reactive oxygen species production, collectively indicating robust enhancement of cellular antioxidant capacity and mitochondrial membrane potential preservation [289]. Mechanistically, melatonin’s therapeutic effects operate through activation of the Nrf2/ARE signaling pathway, as evidenced by significantly increased levels of Nrf2, HO-1, and NQO-1 proteins in injured spinal cord tissue, while concurrently suppressing NLRP3 inflammasome activation through reduced protein expression of NLRP3, ASC, caspase-1, and IL-1β [289]. The critical role of the Nrf2/ARE pathway was confirmed through ML385 inhibitor studies, which reversed melatonin’s protective effects on both oxidative damage and inflammasome suppression [289].

Bi et al. found that melatonin deficiency significantly impedes the natural recovery trajectory of both sensory and motor functions in experimental spinal cord injury models, indicating that adequate melatonin levels are essential for optimal neurological rehabilitation following spinal trauma [290]. Conversely, therapeutic melatonin administration demonstrates remarkable efficacy in accelerating functional recovery through dual mechanisms of neuroprotection and neuroregeneration, specifically by substantially reducing neuronal apoptosis that typically contributes to progressive tissue loss and functional deterioration in the post-injury period [290]. The compound’s therapeutic value extends beyond mere cell survival, as melatonin actively promotes neuronal repair processes that are fundamental to functional restoration and neural circuit reorganization following spinal cord damage [290].

Melatonin exerts profound neuroprotective effects in spinal cord injury through comprehensive modulation of apoptotic pathways and activation of critical regenerative signaling cascades that collectively promote both cellular survival and functional recovery [291]. The compound’s anti-apoptotic mechanisms are demonstrated through significant downregulation of pro-apoptotic proteins Bax and cleaved caspase-3 while simultaneously upregulating the anti-apoptotic protein Bcl-2, creating a favorable cellular environment that promotes neuronal survival following spinal trauma [291]. This shift in apoptotic balance is further validated by the marked reduction in TUNEL-positive cells, indicating decreased DNA fragmentation and programmed cell death in melatonin-treated spinal cord tissue [291]. Beyond cellular preservation, melatonin actively promotes functional recovery by enhancing motor neuronal survival specifically within the spinal cord ventral horn, a critical anatomical region housing motor neurons essential for locomotor function and voluntary movement control [291]. The therapeutic efficacy of melatonin is mechanistically underpinned by its activation of the Wnt/β-catenin signaling pathway, a fundamental cellular communication system known for its neuroprotective and regenerative properties [291]. This activation is evidenced by significant upregulation of key pathway components, including phosphorylated low-density lipoprotein receptor-related protein 6 (p-LRP-6), lymphoid enhancer factor-1 (LEF-1), and β-catenin protein expression in injured spinal cord tissue [291].

### 3.15. Metformin

Metformin, a well-known antidiabetic drug, has garnered attention for its antioxidant capabilities in the context of spinal cord injuries [292]. It demonstrates comprehensive therapeutic efficacy in spinal cord injury through multifaceted neuroprotective mechanisms that encompass functional restoration, cellular preservation, axonal regeneration, and oxidative stress mitigation [293]. Functional assessment using the Basso–Beattie–Bresnahan locomotor rating scale revealed significant improvement in neurological recovery following metformin administration compared to untreated spinal cord injury groups, with histopathological examination confirming substantial protection of both peripheral white matter and central gray matter structures, including critical preservation of anterior horn motor neurons essential for motor function [293]. The compound’s therapeutic effects operate primarily through activation of the PI3K/Akt signaling pathway, as evidenced by the significant suppression of functional recovery benefits when this pathway was pharmacologically inhibited with LY294002, establishing this cascade as fundamental to metformin’s neuroprotective action [293]. Its antiapoptotic properties were demonstrated through marked reduction in apoptotic cell numbers and modulation of key apoptotic regulators, specifically blocking spinal cord injury-induced increases in cleaved caspase-3 and Bax expression while enhancing anti-apoptotic Bcl-2 levels, effects that were reversed upon PI3K/Akt pathway inhibition [293]. Beyond cellular survival, metformin actively promotes axonal regeneration and repair through microtubule stabilization, increasing acetylated tubulin expression indicative of stable microtubules and microtubule-associated protein 2 (MAP2) while reducing tyrosinated tubulin representing dynamic microtubules, resulting in an enhanced acetylated tubulin/tyrosinated tubulin ratio and increased growth cone size that facilitates intrinsic axon growth capacity [293]. The compound’s antioxidative mechanisms involve robust activation of the Nrf2/ARE signaling pathway, significantly upregulating key antioxidant proteins including Nrf2, HO-1, and NQO1 while simultaneously alleviating mitochondrial dysfunction through restoration of mitochondrial membrane potential and cellular ATP levels that are typically compromised following oxidative insult [293]. These convergent mechanisms—PI3K/Akt-mediated neuroprotection, apoptosis suppression, microtubule stabilization for axonal repair, and Nrf2-mediated antioxidant activation—establish metformin as a multi-target therapeutic agent capable of addressing the complex pathophysiological processes underlying spinal cord injury progression and functional impairment.

### 3.16. Omega-3 Fatty Acids

Omega-3 fatty acids demonstrate neuroprotective and antioxidant effects in SCI [294,295]. They mitigate oxidative stress in spinal cord injuries by augmenting the production of endogenous antioxidants, such as carnosine and homocarnosine, and by re-establishing homeostatic levels of oxidative stress markers [294,295,296,297,298].

The prophylactic benefits of omega-3 fatty acids are multifaceted, encompassing neuronal cell membrane stabilization coupled with potent anti-inflammatory effects evidenced by significant reduction in key inflammatory mediators, including IL-6, KC/GRO/CINC, IL-1ra, C-reactive protein, and TNF-α, with genetically modified fat-1 mice demonstrating accelerated recovery trajectories accompanied by diminished inflammatory cytokine expression and reduced macrophage infiltration [298]. These compounds enhance local microvascular perfusion while simultaneously reducing eicosanoid production, creating a favorable microenvironment for neural repair, and activate critical protective intracellular transcription pathways involving RXR, PPAR-α, Akt, and CREB signaling cascades that promote cellular survival and regeneration [298]. Omega-3 fatty acids also augment neuronal energy metabolism by increasing concentrations of essential cellular substrates including lipids, glycogen, and oligosaccharides, while their robust antioxidant properties manifest through enhanced endogenous antioxidant production such as carnosine and homocarnosine, reduced oxidative stress markers like malondialdehyde (MDA), and maintained glutathione (GSH) concentrations at injury sites, collectively limiting neutrophil infiltration and inhibiting neuronal apoptosis [298]. Therapeutically, omega-3 fatty acids facilitate motor function recovery through enhanced autophagy mechanisms evidenced by increased LC3-II expression and improved Basso–Beattie–Bresnahan locomotor scores while simultaneously reducing neuropathic pain through decreased p38 MAPK expression in superficial dorsal horns [298]. The compounds’ antiapoptotic properties are demonstrated through upregulation of neuroprotective gene expression and enhanced cellular resistance to glutamate toxicity, apoptosis, and calcium overload, accompanied by reduced expression of proapoptotic proteins including p53 and caspase-3 [298].

Bi et al. found that omega-3 fatty acids have potent antioxidative properties, as evidenced by complete normalization of key oxidative stress markers, including restoration of lipid peroxidation levels and rebalancing of critical antioxidant enzyme systems comprising reduced glutathione, superoxide dismutase, glutathione peroxidase, and catalase, thereby reestablishing the cellular redox homeostasis that is typically disrupted following spinal cord trauma [295]. Omega-3 supplementation exerts remarkable anti-inflammatory effects, achieving greater than 50% reduction in both tumor necrosis factor-alpha (TNF-α) and interleukin-6 (IL-6) protein levels, with corresponding decreases in mRNA expression, indicating comprehensive suppression of pro-inflammatory cascades at both transcriptional and translational levels that contribute to secondary injury progression [295]. The compound’s antiapoptotic mechanisms operate through dramatic modulation of key cell death regulators, effectively counteracting the spinal cord injury-induced upregulation of pro-apoptotic factors, including caspase-3, p53, bax, and pro-NGF mRNA expression by over 40%, while simultaneously enhancing anti-apoptotic bcl-2 mRNA expression by a remarkable 286.9%, creating a cellular environment strongly favoring survival over programmed cell death [295]. These transcriptional changes translate into significant protein-level modifications, with omega-3 supplementation reducing caspase-3 and p53 protein expression by more than 30%, confirming the functional impact of these molecular alterations on cellular fate determination [295].

### 3.17. Quercetin

Quercetin, a naturally occurring flavonoid, presents itself as a promising candidate for mitigating the effects of spinal cord injuries due to its observed antioxidant and neuroprotective properties [299,300,301,302]. Its capacity to alleviate oxidative stress and inflammation, coupled with its potential to encourage neural regeneration, positions it as a viable option for therapeutic intervention in SCI [300,301,302].

Quercetin demonstrates equivalent therapeutic efficacy to methylprednisolone in experimental spinal cord injury treatment [303]. Biochemical analysis reveals that spinal cord injury induces significant oxidative stress. This manifests as elevated plasma and tissue levels of nitric oxide (NO) and malondialdehyde (MDA) coupled with reduced total antioxidant levels (TAL) [303]. Both single- and multiple-dose quercetin regimens effectively counteract these pathological changes [303]. The treatments achieve statistically significant normalization of oxidative markers comparable to methylprednisolone outcomes [303]. Functional assessment using inclined-plane testing demonstrates significant improvements with all quercetin treatment protocols [303]. Motor coordination and postural stability improve compared to untreated trauma groups, with therapeutic benefits equivalent to those achieved with methylprednisolone [303]. However, the relatively brief 72 h treatment duration may have limited detection of more comprehensive motor function improvements [303]. Focused clinical examinations assess individual extremity function rather than integrated motor performance [303]. Histopathological examination reveals that quercetin treatment effectively preserves spinal cord architecture [303]. The compound completely eliminates inflammatory infiltration while increasing multipolar motor neuron survival [303]. Glial cell distribution normalizes, and post-traumatic edema and neutrophil infiltration are reduced [303]. Some differences emerge between treatment modalities [303]. Methylprednisolone shows superior edema reduction in myelinated nerve fibers, whereas quercetin-treated groups demonstrate increased edema in these structures despite overall tissue preservation [303]. Quercetin’s tissue-protective effects extend to restoration of grey and white matter integrity [303]. The treatment prevents canalis centralis and ependymal cell disruption that characterizes untreated spinal cord injury [303].

Biochemical analysis revealed that quercetin treatment substantially enhances total antioxidant capacity (TAS) and paraoxonase-1 (PON-1) activity levels in both serum and spinal cord tissue compared to untreated trauma groups, with the most pronounced benefits observed when quercetin is administered in combination with resveratrol, indicating synergistic antioxidative effects that exceed the therapeutic potential of either compound alone [304]. Histological examination demonstrates quercetin’s anti-inflammatory properties through significant reduction of polymorphonuclear leukocyte (PMNL) infiltration in injured spinal cord tissue, comparable to resveratrol treatment outcomes, while exhibiting superior efficacy in attenuating microglia, macrophage, and mononuclear leukocyte (MNL) infiltration in both white and gray matter regions, which represents a critical therapeutic advantage given the central role of neuroinflammatory cell infiltration in secondary injury progression [304]. Quercetin also contributes to vascular stabilization and tissue preservation by reducing post-traumatic hemorrhage, though resveratrol demonstrates superior hemorrhage control, while both compounds effectively ameliorate edema formation, suggesting their collective utility in addressing post-traumatic oxidative stress and vascular disruption [304]. However, quercetin’s therapeutic profile reveals limitations in addressing certain aspects of spinal cord pathology, specifically showing no beneficial effect on axonal swelling induced by spinal cord injury, whether administered alone or in combination with resveratrol, and demonstrating only non-significant improvement trends in chromatolysis reduction [304].

Electrophysiological testing confirms enhanced neural conduction with quercetin administration, reducing latency while increasing amplitude of both somatosensory evoked potentials (SEP) and motor evoked potentials (MEP) [305]. These functional improvements correlate with marked structural preservation demonstrated through hematoxylin–eosin staining [305]. Quercetin treatment significantly decreases lesion size and cavity formation in injured spinal cord tissue [305]. Biotinylated dextran amine anterograde tracing reveals increased BDA-positive fibers following quercetin treatment, indicating enhanced axonal regeneration capacity [305]. Immunofluorescence analysis demonstrates quercetin’s ability to elevate 5-hydroxytryptamine (5-HT)-positive nerve fibers and neurofilament-200 (NF-200)-positive neurons [305]. The compound simultaneously reduces glial fibrillary acidic protein (GFAP)-positive astrocytes and inhibits GFAP expression while increasing both NeuN and NF-200 expression [305]. This suggests effective modulation of glial scarring and neuronal preservation [305]. Quercetin facilitates spinal cord energy metabolism through enhanced glucose utilization, which is evidenced by increased 18F-FDG uptake in a time-dependent manner, indicating improved metabolic activity essential for tissue repair [305]. The compound’s neuroprotective mechanisms involve autophagy induction through increased expression of Beclin 1 and LC3 II markers [305]. Quercetin modulates the Akt/mTOR/p70S6K signaling pathway by blocking phosphorylation of these key regulatory proteins [305]. The critical role of autophagy in quercetin’s therapeutic effects is confirmed by partial abolition of neuroprotective benefits when autophagy is inhibited with 3-methyladenine [305].

In the study by Wang et al., quercetin significantly enhanced motor function recovery, with improved Basso–Beattie–Bresnahan scores observed consistently at 1, 3, 5, and 7 days post-injury compared to untreated controls [306]. Electrophysiological evaluation further confirms quercetin’s therapeutic benefits, with enhanced somatosensory evoked potentials (SEPs) and motor evoked potentials (MEPs) recordings at 7 days post-injury, indicating improved neural conduction pathways essential for functional recovery [306]. Histopathological examination using hematoxylin–eosin staining demonstrates that quercetin effectively reduces cavity formation in neural tissue following acute spinal cord injury, preserving structural integrity crucial for maintaining neural connectivity [306]. The compound’s regenerative properties are evidenced through enhanced cellular responses, including quercetin-mediated astrocyte activation, as demonstrated by immunohistochemistry staining for glial fibrillary acidic protein (GFAP) and increased expression of both GFAP and S100β proteins [306]. Quercetin actively promotes axonal regeneration, with enhanced axonal growth confirmed through specific antibody staining for neurofilament 200 (NF200) and 5-hydroxytryptamine (5-HT), indicating restored neural connectivity and neurotransmitter pathway integrity [306]. The compound’s molecular mechanisms involve strategic protein expression modulation, specifically upregulating brain-derived neurotrophic factor (BDNF) expression, which is critical for neuronal survival, growth, and synaptic plasticity [306]. Simultaneously, quercetin reduces expression of phosphorylated JNK2 (p-JNK2) and phosphorylated STAT3 (p-STAT3) following acute spinal cord injury, suggesting effective modulation of stress-activated signaling pathways that typically contribute to secondary tissue damage and impaired recovery outcomes [306].

Fan et al. found that quercetin’s primary neuroprotective mechanism involves prevention of oligodendrocyte necroptosis rather than apoptosis, which represented the predominant cell death pathway in their experimental model [307]. Quercetin significantly reduces necroptotic oligodendrocyte loss, as evidenced by decreased numbers of RIP3/CC1, MLKL/CC1, and pMLKL/CC1 double-positive cells that serve as specific necroptosis markers [307]. In vitro validation demonstrates that quercetin effectively inhibits oligodendrocyte necroptosis induced by pro-inflammatory M1 macrophages/microglia through reduced intracellular reactive oxygen species levels, increased ATP production, and decreased propidium iodide labeling [307]. The compound also suppresses expression of necroptotic markers, including RIP3, MLKL, and p-MLKL, when oligodendrocytes are exposed to M1-conditioned medium [307]. Quercetin’s tissue-protective effects extend to significant preservation of myelin and axonal integrity following spinal cord injury [307]. Luxol fast blue staining and immunostaining reveal attenuated reduction of MBP-positive myelin and NF200-positive axons in quercetin-treated animals compared to controls [307]. Electron microscopic analysis confirms that quercetin treatment prevents spinal cord injury-induced myelin sheath decompaction, maintains lower g-ratios, and preserves axonal numbers [307]. The compound strategically modulates macrophage/microglia polarization by suppressing pro-inflammatory M1 phenotype activation, evidenced by decreased mRNA levels of M1 markers, including TNFα, iNOS, and CD86, alongside reduced iNOS-expressing cell populations [307]. Simultaneously, quercetin promotes beneficial M2 phenotype polarization through increased mRNA expression of anti-inflammatory markers such as Arginase1, IL-4, and CD206 [307]. These polarization effects are mediated through inhibition of STAT1 and NF-κB signaling pathways, with quercetin significantly reducing expression of iNOS, pSTAT1, NF-κB, and p-NF-κB both in vitro and in vivo, establishing a comprehensive therapeutic approach that addresses multiple pathological processes underlying spinal cord injury progression [307].

### 3.18. Resveratrol

Resveratrol, a naturally occurring polyphenolic compound, has garnered significant attention for its therapeutic potential in the context of spinal cord injuries, largely attributable to its antioxidant properties [308,309,310]. Research indicates that resveratrol may play a role in alleviating oxidative stress, diminishing inflammation, and facilitating functional recovery in diverse experimental models of SCI [88,309,310,311].

Resveratrol demonstrates significant therapeutic efficacy in spinal cord injury recovery through targeted inhibition of ferroptosis [310]. Motor function assessment using the Basso Mouse Scale score and footprint analysis reveals that resveratrol treatment substantially improves locomotor recovery in experimental spinal cord injury models compared to untreated controls [310]. The compound exhibits notable neuroprotective properties that extend beyond motor function enhancement, providing comprehensive neural tissue preservation in the acute and subacute phases following spinal cord injury [310]. Resveratrol’s primary therapeutic mechanism involves effective inhibition of ferroptosis through suppression of ferroptosis-related protein expression and modulation of iron homeostasis that typically becomes dysregulated after spinal trauma [310]. The compound also ameliorates mitochondrial morphological alterations that characterize ferroptotic cell death, preserving cellular energy production capacity essential for neural repair and survival [310]. Mechanistic investigation establishes that resveratrol’s anti-ferroptotic effects operate through activation of the Nrf2/GPX4 signaling pathway, a critical cellular defense system against lipid peroxidation and oxidative damage [310]. This pathway dependence is confirmed through pharmacological validation, where the Nrf2 inhibitor ML385 completely reverses resveratrol’s inhibitory effects on ferroptosis-related gene expression, demonstrating the specificity of this therapeutic mechanism [310]. The Nrf2/GPX4 pathway activation enables enhanced antioxidant enzyme production and lipid peroxide neutralization, effectively preventing the accumulation of toxic lipid peroxidation products that drive ferroptotic cell death in injured neural tissue [310].

Resveratrol strategically inhibits NF-κB signaling pathway activation while simultaneously reducing ATP production and reactive oxygen species levels, thereby preventing NLRP2 inflammasome formation in astrocytes that would otherwise perpetuate neuroinflammatory cascades [309]. It effectively suppresses expression of pro-inflammatory factors and cytokines that contribute to secondary injury progression and tissue destruction following spinal cord trauma [309]. A critical therapeutic benefit involves resveratrol’s ability to mitigate glial scarring through attenuation of astrocyte activation and inflammatory response inhibition, which is essential for enhancing axonal recovery and overall spinal cord repair processes [309]. The compound modulates immune responses by interfering with pattern recognition receptor (PRR) recognition of damage-associated molecular patterns, thereby decreasing inflammasome formation that would otherwise amplify tissue damage [309]. Resveratrol also downregulates Toll-like receptor (TLR) expression, which plays a pivotal role in activating inflammatory pathways that impede neural recovery and promote secondary degeneration [309].

In the study by Wang et al., motor function assessment reveals that resveratrol treatment significantly enhances hindlimb locomotor performance in spinal cord injury models, with elevated BBB scores and inclined plane test results observed consistently from 7 to 28 days post-injury compared to untreated controls [312]. Histological analysis confirms that resveratrol substantially increases the number of preserved motor neurons in the spinal cord anterior horn, indicating effective prevention of motor neuron loss that typically occurs following spinal trauma [312]. The compound’s anti-apoptotic properties are evidenced through significant reduction of cleaved caspase-3 and Bax expression levels, coupled with increased Bcl-2 expression and decreased Bax/Bcl-2 ratio in resveratrol-treated animals [312]. Immunofluorescence staining further validates these findings by demonstrating markedly reduced numbers of cleaved caspase-3-positive neurons in resveratrol-treated groups compared to injury controls [312]. Resveratrol effectively ameliorates neuronal autophagic flux obstruction that characterizes spinal cord injury pathophysiology [312]. The compound significantly elevates the LC3-II/I ratio, indicating enhanced autophagosome formation and autophagic activity essential for cellular debris clearance and survival [312]. Simultaneously, resveratrol reduces p62 protein levels, which typically accumulate when autophagic degradation is impaired, confirming restoration of efficient autophagic flux [312]. The critical role of autophagy in resveratrol’s therapeutic mechanism is demonstrated through pharmacological validation, where chloroquine phosphate-mediated autophagy blockade completely eliminates resveratrol’s neuroprotective effects, including anti-apoptotic actions and motor function improvements [312]. Mechanistically, resveratrol activates the LKB1/AMPK/mTOR/p70s6k signaling pathway both in vivo and in vitro, as evidenced by increased LKB1 and phosphorylated AMPK levels alongside decreased phosphorylated mTOR and p70s6k ratios [312]. This pathway serves as the primary mediator of resveratrol-induced autophagic flux activation, with pathway inhibition or LKB1 suppression abolishing the compound’s therapeutic benefits and confirming the mechanistic dependence on this cellular energy-sensing cascade for optimal neuroprotective outcomes [312].

Rats treated with resveratrol exhibited enhanced recovery of locomotor activity, as reflected in higher Basso–Beattie–Bresnahan scores and better performance in the inclined plane test compared to untreated SCI groups [88]. These functional improvements are consistent with resveratrol’s established neuroprotective properties, including its ability to preserve neuronal integrity and support neurological recovery [88]. In addition to its neuroprotective effects, resveratrol demonstrated potent anti-inflammatory properties [88]. Treatment with resveratrol led to a marked reduction in pro-inflammatory cytokines, such as tumor necrosis factor-α (TNF-α) and interleukin-1β (IL-1β), while simultaneously increasing levels of the anti-inflammatory cytokine interleukin-10 (IL-10) at the injury site [88]. These findings indicate that resveratrol effectively attenuates the neuroinflammatory response triggered by spinal trauma [88]. Moreover, resveratrol was found to enhance autophagy, a key cellular process involved in the degradation and recycling of damaged cellular components [88]. Following SCI, autophagy was activated, as indicated by elevated LC3-II/LC3-I ratios and increased Beclin-1 expression [88]. Resveratrol treatment further amplified these autophagic markers, an effect linked to modulation of the AMPK/mTOR signaling pathway [88]. Specifically, it increased phosphorylated AMPK and decreased phosphorylated mTOR levels, suggesting activation of autophagy through this pathway [88]. Importantly, the neuroprotective effects of resveratrol were diminished when autophagy was pharmacologically inhibited using 3-methyladenine (3-MA), supporting the conclusion that autophagy activation is a critical mechanism underlying resveratrol’s therapeutic benefits in SCI [88].

Resveratrol has demonstrated significant anti-inflammatory properties and the ability to suppress the NF-κB pathway, which is critically involved in the pathogenesis of SCI [313]. It reduces injury-induced myeloperoxidase activity, decreases levels of pro-inflammatory cytokines such as TNF-α and IL-10, and limits neutrophil infiltration at the injury site [313]. Furthermore, it downregulates the expression of several NF-κB-mediated inflammatory mediators, including TGF-β, MMPs, COX-2, IL-1β, IL-6, and ICAM-1, thereby disrupting the cascade of secondary inflammation [313]. In addition to its anti-inflammatory effects, resveratrol enhances cellular antioxidant defenses. It increases the activity of superoxide dismutase and reduces malondialdehyde levels, mitigating oxidative stress and protecting cells from free radical-induced damage [313]. Its anti-apoptotic actions are equally critical, as it promotes the expression of Bcl-2, an anti-apoptotic protein, while suppressing pro-apoptotic markers such as Bax and caspase-3, thereby reducing neuronal cell death following SCI [313]. Moreover, resveratrol modulates key intracellular signaling pathways, particularly the SIRT1-AMPK axis [313]. This pathway plays a vital role in regulating autophagy and apoptosis, contributing to resveratrol’s neuroprotective effects [313]. It also inhibits the phosphorylation of IκB and reduces nuclear levels of the p65 subunit, leading to decreased NF-κB transcriptional activity and further suppression of inflammation [313].

### 3.19. Tetramethylpyrazine

Tetramethylpyrazine (TMP), a bioactive compound derived from the Chinese herb Ligusticum wallichii, exhibits multiple mechanisms of action, including anti-inflammatory, anti-apoptotic, and antioxidant properties, which contribute to its therapeutic potential in SCI [314,315,316,317,318].

**Table 2:** Antioxidants in spinal cord injury.

Compound	Mechanism of Action	Therapeutic Effects	References
A91 peptide	Immunomodulatory properties; reduces nitric oxide production; downregulates iNOS gene expression; enhances neurotrophic factor production (BDNF, NT-3)	Neuroprotective effects; anti-inflammatory action; enhanced functional recovery in moderate SCI (injury severity-dependent)	[229,230,231,232,233]
Allicin	Antioxidant properties; anti-inflammatory effects via NF-κB and TNF-α reduction; upregulates HSP70/Akt/iNOS signaling; attenuates glutamate-induced excitotoxicity	Enhanced functional recovery; reduced spinal cord edema; neuroprotection against oxidative stress and excitotoxicity	[234,235]
Asiatic acid/asiaticoside	Anti-inflammatory and antioxidant properties; reduces lipid peroxidation; suppresses pro-inflammatory cytokines; modulates apoptotic cascades	Improved motor function recovery; reduced tissue damage; enhanced neuronal survival and structural preservation	[236,237]
Curcumin	Modulates Nrf2, NF-κB, and TGF-β pathways; enhances autophagy; inhibits Akt/mTOR signaling; activates ERK1/2 pathway; epigenetic regulation via miR-137-3p/NeuroD1	Comprehensive neuroprotection; reduced inflammation and apoptosis; enhanced tissue integrity and functional recovery; superior long-term efficacy	[238,239,240,241,242,243,244,245,246,247,248,249,250,251,252,253]
Edaravone	Ferroptosis pathway regulation; upregulates anti-ferroptosis proteins (GPX4); anti-inflammatory effects; enhances BMSC differentiation into neurons	Enhanced neuronal survival; improved tissue sparing; superior functional recovery and neural regeneration	[255,256,257,258,259]
Epigallocatechin gallate (EGCG)	Antioxidant effects; inhibits p38MAPK/NF-κB/AQP4 signaling; reduces inflammatory mediators; anti-edema properties	Reduced oxidative stress; effective anti-inflammatory and anti-edema effects; tissue preservation	[260,261,262]
Estrogen	Dose-dependent neuroprotection; modulates inflammatory and apoptotic pathways; preserves neuronal survival; anti-inflammatory via COX-2 inhibition	Enhanced survival rates; superior locomotor function; reduced tissue damage and inflammatory response	[263,264,265,266]
Gastrodin	Antioxidant and anti-inflammatory properties; preserves blood–spinal cord barrier; upregulates Nrf2-GCLc/GCLm signaling	Improved locomotor function; reduced inflammatory cytokines; enhanced antioxidant capacity	[267]
Ginkgo biloba extract 761 (EGb761)	Antioxidant activity; modulates apoptotic genes (Bcl-2/Bax ratio); inhibits cPLA2 and ERK1/2 signaling; reduces free radical generation	Functional and histopathological improvements; reduced apoptosis and tissue necrosis; neuroprotection against oxidative damage	[268,269,270,271,272]
Ginsenosides	Immunomodulatory via miR-130b-5p/TLR4 pathway; autophagy inhibition; anti-inflammatory and antioxidant effects; MAPK pathway inhibition	Reduced neuronal apoptosis; enhanced motor function recovery; tissue preservation, and reduced inflammatory response	[273,274,275,276,277]
Glutathione	Antioxidant defense; chirality-dependent effects (D-GSH superior); MAPK pathway modulation; age-related therapeutic variations	Enhanced axon regeneration; improved motor function recovery; age-specific therapeutic efficacy	[36,194,278,279]
Ligustilide	Anti-inflammatory and antioxidative effects; suppresses inflammatory mediators (iROS, PGE2, IL-1β, TNF-α); downregulates iNOS	Enhanced motor function recovery; reduced inflammatory and oxidative damage	[280,281]
Lycopene	Blood–spinal cord barrier stabilization; anti-inflammatory via TNF-α and NF-κB suppression; upregulates tight junction proteins; antioxidative properties	Improved motor function; reduced spinal cord edema; enhanced barrier integrity and tissue preservation	[282,283]
Melatonin	Antioxidant effects; activates Nrf2/ARE pathway; suppresses NLRP3 inflammasome; modulates Wnt/β-catenin signaling; anti-apoptotic mechanisms	Enhanced motor function recovery; increased neuronal survival; reduced oxidative stress and inflammation	[284,285,286,287,288,289,290,291]
Metformin	PI3K/Akt pathway activation; Nrf2/ARE signaling; microtubule stabilization; antiapoptotic effects; antioxidative mechanisms	Comprehensive neuroprotection; enhanced axonal regeneration; improved functional recovery and cellular preservation	[292,293]
Omega-3 fatty acids	Antioxidant effects via endogenous antioxidant production; anti-inflammatory action; neuronal membrane stabilization; activates protective transcription pathways (RXR, PPAR-α, Akt, CREB)	Neuroprotective and antioxidant effects; enhanced functional recovery; reduced inflammatory response and apoptosis	[294,295,296,297,298]
Quercetin	Antioxidant and anti-inflammatory properties; modulates Akt/mTOR/p70S6K signaling; prevents oligodendrocyte necroptosis; induces autophagy	Enhanced motor function recovery; preserved neural tissue; improved myelin integrity and axonal regeneration	[299,300,301,302,303,304,305,306,307]
Resveratrol	Inhibits ferroptosis via Nrf2/GPX4 pathway; suppresses NF-κB signaling; activates LKB1/AMPK/mTOR autophagy pathway; anti-inflammatory effects	Improved locomotor recovery; reduced neuronal apoptosis; enhanced autophagy and tissue preservation	[88,308,309,310,311,312,313]
Tetramethylpyrazine	Anti-inflammatory, anti-apoptotic, and antioxidant properties; upregulates PGC-1α; reduces glial scar formation; preserves neuronal structure	Enhanced motor function recovery; reduced inflammation and glial scarring; improved neuronal survival	[314,315,316,317,318]

In the study by Li et al., tetramethylpyrazine demonstrated significant neuroprotective effects in a spinal cord injury mouse model, particularly through improvements in motor function, reduction of inflammation, and preservation of spinal tissue integrity [318]. Mice treated with a high dose (100 mg·kg^−1^) of tetramethylpyrazine showed markedly improved hind limb motor function, as evidenced by significantly higher Basso Mouse Scale and inclined plate test scores compared to the untreated model group (*p* < 0.01) [318]. In addition to functional recovery, tetramethylpyrazine treatment significantly reduced inflammatory responses within the injured spinal cord [318]. The levels of pro-inflammatory cytokines, including TNF-α, IL-6, and IL-1β, were notably decreased in the high-dose group (*p* < 0.01), suggesting strong anti-inflammatory activity [318]. Histological analysis further revealed improved spinal cord morphology, with an increase in the number and structural integrity of Nissl bodies, indicating a reduction in neuronal damage and enhanced neuronal preservation [318]. Moreover, tetramethylpyrazine treatment led to a significant decrease in glial scar formation markers [318]. Specifically, the expression levels of glial fibrillary acidic protein (GFAP) and complement component C3, both associated with astrocyte activation and glial scarring, were significantly reduced in the high-dose group compared to the model group (*p* < 0.05, *p* < 0.01) [318].

Rats treated with TMP exhibited markedly higher Basso–Beattie–Bresnahan locomotor scores compared to those receiving normal saline (NS), particularly from day 7 to day 28 post-injury, indicating enhanced locomotor function recovery [314]. The most rapid phase of improvement occurred between days 3 and 7, highlighting this period as a critical therapeutic window in which TMP exerted its strongest effect [314]. At the molecular level, the expression of peroxisome proliferator-activated receptor gamma coactivator-1 alpha (PGC-1α), a key regulator of mitochondrial biogenesis and neuroprotection, was found to decrease significantly—by approximately 80%—within 24 h of injury [314]. However, TMP treatment effectively reversed this trend, significantly upregulating PGC-1α expression at several time points post-injury (3, 7, 21, and 28 days), compared to the NS group [314]. Although overall expression did not return to baseline levels, the increase correlated with ongoing repair processes. Importantly, PGC-1α was primarily localized in the grey matter of the spinal cord and co-localized with neurons, especially in the ventral horn, a pattern that persisted even after injury despite evident structural damage [314]. TMP also demonstrated neuroprotective effects by reducing neural apoptosis and promoting neuronal survival [314]. At day 7 post-injury, TUNEL staining showed a significantly lower percentage of apoptotic cells in the TMP group (approximately 11%) compared to the NS group (28%) [314]. Complementary Nissl staining revealed a higher number of morphologically intact Nissl-positive neurons in the TMP-treated animals, suggesting improved preservation of neuronal structure and function [314].

## 4. Novel Formulations and Delivery Systems

The development of novel formulations and delivery systems represents a critical advancement in spinal cord injury treatment, as conventional antioxidant administration faces significant barriers, including poor bioavailability, limited tissue penetration, and rapid clearance from the injury site. These delivery challenges are particularly pronounced in spinal cord injury due to the blood–spinal cord barrier disruption, inflammatory responses, and the need for sustained therapeutic concentrations at the precise injury location during the critical acute phase. Advanced delivery systems offer the potential to overcome these limitations by providing targeted, controlled, and prolonged antioxidant release directly to damaged neural tissue, thereby maximizing therapeutic efficacy while minimizing systemic side effects (Table 3).

**Table 3:** Novel antioxidant formulations and delivery systems for spinal cord injury treatment.

Compound	Delivery System	Key Properties	Mechanisms of Action	In Vitro Results	In Vivo Results	References
Curcumin	Nanocomposite with resveratrol in calcium alginate hydrogel	Ionotropic gelation-based platform; sustained release kinetics	Downregulation of NF-κB and TNF-α gene expression; anti-inflammatory effects	Complete absence of cytotoxicity against PC-12 neuronal cells; sustained release of both compounds	Superior healing outcomes in rat SCI model; effective modulation of inflammatory signaling cascades	[319]
Curcumin	Curcumin nanoconjugate (PA-C)	Dose-dependent enhancement above 10 µM without cytotoxicity	Prevention of H_2_O_2_-induced cytotoxicity; reduction of LPS-induced NF-κB translocation	Enhanced iPSC-derived neural stem cell viability; promoted neurite elongation in β-III tubulin-positive cells	No significant BBB scale improvements, but reduced glial scar area; enhanced β-III tubulin preservation; promoted M2 microglial polarization	[320]
EGCG	EGCG-selenium nanoparticles (EGCG-Se NP)	Rapid ROS scavenging capacity	Dual antioxidant and anti-inflammatory mechanisms	Protected PC12 cells from H_2_O_2_-induced oxidative damage	Significant locomotor capacity improvements; substantial reduction in injury area; protection of neuronal cell bodies and myelin sheaths	[261]
Estrogen	Estrogen nanoparticles with engineered release kinetics	Fast-release and slow-release variants; enhanced tissue distribution	Modulation of inflammatory responses, apoptotic signaling, and tissue preservation	Reduced ROS production and calpain activity in microglia, astroglia, macrophages, and fibroblasts	Fast release: reduced Bax/Bcl-2 ratio; slow release: prevented gliosis and penumbral demyelination	[321]
Metformin	Glutathione-modified macrophage-derived cell membrane-encapsulated nanogels (Met-CNG-GSH)	Biomimetic cell membrane coating; glutathione modification for BSCB penetration	Addresses oxidative stress, neuroinflammation, and apoptotic cell death	Optimal sustained-release characteristics	Significant accumulation at injury sites; amelioration of oxidative stress, neuroinflammation, and apoptosis	[322]
Resveratrol	Chitosan-modified hollow manganese dioxide nanoparticles (CMR)	~130 nm particle size; 21.39 ± 2.53% drug loading efficiency	Antioxidative, anti-inflammatory, and anti-apoptotic effects	87% sustained release over 36 h	Reduced ROS, MDA, and SOD levels; increased GPx activity; reduced iNOS, IL-1β expression; downregulated Cl caspase-3, Bax; upregulated Bcl-2	[323]
Resveratrol + Puerarin	Polymeric nanoparticles (RES-PUE)	238–274 nm particle size; −12.6 ± 2.1 mV zeta potential; 74.85% encapsulation efficiency	Addresses inflammation and neuronal apoptosis	72–79% sustained release over 36 h vs. 96–98% rapid release of native drugs within 6 h	Decreased MDA and AOPP levels; reduced plasma nitrite/nitrate; normalized iNOS expression; increased SOD and catalase activity	[324]
Tetramethylpyrazine (TMP)	HIV TAT-modified nanoparticles (TAT-TMP-NPs)	163.93 ± 0.38 nm size; −30 mV surface charge; 77.27 ± 1.99% encapsulation efficiency	Enhanced blood–spinal cord barrier penetration and targeting	80–82% sustained release over 96 h; >80% cell viability at high concentrations; <5% hemolysis rate	Enhanced targeting to spinal cord tissue; improved bioavailability and extended circulation time	[325]
Tetramethylpyrazine (TMP)	Electroconductive hydrogel	Integrated approach combining drug delivery with tissue engineering	Targets microvascular dysfunction and neural regeneration simultaneously	Enhanced pharmacological effectiveness	Supports synergistic tissue repair environment; addresses vascular stabilization and neural regeneration	[326]

### 4.1. Curcumin

The developed nanocomposite delivery system represents a significant advancement in spinal cord injury therapeutics through the coordinated delivery of curcumin and resveratrol via an innovative ionotropic gelation-based platform dispersed within a calcium alginate hydrogel matrix [319]. In vitro characterization confirmed the biocompatibility and functional efficacy of the system, demonstrating complete absence of cytotoxicity against PC-12 neuronal cells while achieving sustained release kinetics for both therapeutic compounds, ensuring prolonged bioavailability and therapeutic exposure at the injury site [319]. In vivo evaluation utilizing a rat spinal cord injury model revealed that co-administration of curcumin and resveratrol through this nanocomposite system produced significantly superior healing outcomes compared to alternative treatment approaches, establishing the synergistic therapeutic potential of this dual-compound delivery strategy [319]. The enhanced therapeutic efficacy was mechanistically attributed to effective modulation of inflammatory signaling cascades, specifically through downregulation of nuclear factor kappa B (NF-κB) and tumor necrosis factor-alpha (TNF-α) gene expression profiles, indicating targeted suppression of key inflammatory pathways that drive secondary injury progression and limit recovery potential [319]. This nanocomposite platform addresses critical challenges in spinal cord injury treatment by providing controlled, sustained delivery of complementary neuroprotective compounds while directly targeting fundamental inflammatory mechanisms that determine injury severity and functional outcomes [319]. The integration of biocompatible nanotechnology with synergistic therapeutic agents represents a promising translational approach that enhances drug delivery efficiency, prolongs therapeutic exposure, and optimizes anti-inflammatory effects, positioning this system as a viable clinical intervention for improving spinal cord injury management and recovery outcomes [319].

Curcumin nanoconjugate (PA-C) demonstrates significant neuroprotective potential in spinal cord injury treatment through comprehensive in vitro mechanisms, though translation to functional recovery remains limited despite substantial histological improvements [320]. In vitro evaluation revealed that PA-C treatment enhances induced pluripotent stem cell-derived neural stem cell viability and metabolic activity in a dose-dependent manner above 10 µM concentrations without cytotoxicity while promoting significant neurite elongation in β-III tubulin-positive cells, though it failed to counteract lysophosphatidic acid-induced neurite retraction, unlike established Rho kinase inhibitors [320]. The compound’s neuroprotective properties were evidenced by effective prevention of hydrogen peroxide-induced cytotoxicity at concentrations of 5–15 µM, addressing oxidative stress that drives secondary injury cascades, while anti-inflammatory effects were demonstrated through significant reduction of lipopolysaccharide-induced nuclear factor-κB translocation, a critical pro-inflammatory mediator in spinal cord injury pathophysiology [320]. Despite these promising cellular effects, PA-C treatment did not significantly induce neural differentiation or alter astrocyte-to-neuron ratios in stem cell populations [320]. In vivo translation revealed a complex therapeutic profile where PA-C treatment, alone or combined with stem cell therapies, failed to produce statistically significant locomotor function improvements on the BBB scale in contusive spinal cord injury models, though treated animals showed continuous improvement trends over time [320]. However, substantial histological benefits were observed, including a significant reduction in glial scar area—a critical barrier to axonal regrowth—along with enhanced preservation of β-III tubulin-positive neuronal fibers, increased functional synapse numbers, and improved white matter sparing, particularly when combined with iPSC-NSC and mesenchymal stem cell transplantation [320]. Additionally, PA-C treatment promoted beneficial microglial polarization toward anti-inflammatory M2 phenotypes, as evidenced by increased IBA1+Arg1+ microglia at injury sites [320]. These findings establish PA-C as a promising adjuvant therapy that provides substantial tissue-level neuroprotection and creates a more favorable microenvironment for recovery, though the disconnect between histological improvements and functional outcomes highlights the complex challenges in translating cellular benefits to meaningful locomotor recovery in spinal cord injury treatment [320].

### 4.2. Epigallocatechin-3-Gallate

Epigallocatechin-3-gallate selenium nanoparticles (EGCG-Se NP) demonstrate comprehensive neuroprotective efficacy in spinal cord injury through coordinated antioxidant and anti-inflammatory mechanisms that translate from cellular protection to functional recovery outcomes [261]. In vitro evaluation confirmed the compound’s potent antioxidant capacity, with EGCG-Se NP rapidly scavenging excess reactive oxygen species and effectively protecting PC12 neuronal cells from hydrogen peroxide-induced oxidative damage, establishing its cytoprotective properties against oxidative stress that characterizes secondary injury cascades [261]. Translation to in vivo spinal cord injury models revealed significant therapeutic benefits, with intravenous administration of EGCG-Se NP producing marked improvements in locomotor capacity in treated rats compared to controls, demonstrating meaningful functional recovery outcomes [261]. The neuroprotective effects extended to tissue preservation, with EGCG-Se NP treatment substantially reducing injury area through coordinated protection of both neuronal cell bodies and myelin sheaths, critical components for maintaining neural connectivity and signal transmission [261]. Mechanistic analysis confirmed that the primary therapeutic effects of EGCG-Se NP are mediated through its dual capacity for reactive oxygen species scavenging and anti-inflammatory activity, addressing two fundamental pathophysiological processes that drive secondary injury progression and limit recovery potential [261].

### 4.3. Estrogen

Estrogen nanoparticle delivery systems demonstrate superior neuroprotective efficacy in spinal cord injury through targeted modulation of inflammatory responses, apoptotic signaling, and tissue preservation mechanisms that address both acute and chronic pathological processes [321]. In vitro evaluation revealed that estrogen treatment effectively reduced reactive oxygen species production and calpain activity across multiple cell types critically involved in secondary injury progression, including microglia, astroglia, macrophages, and fibroblasts, indicating comprehensive anti-inflammatory and cytoprotective effects that target the cellular mediators of neuroinflammation and glial scar formation [321]. Translation to in vivo spinal cord injury models demonstrated that focal nanoparticle-mediated estrogen delivery achieved enhanced tissue distribution and sustained bioavailability over time, resulting in significant attenuation of cell death and improved myelin preservation within the injured spinal cord compared to conventional delivery approaches [321]. The therapeutic benefits were further optimized through engineered release kinetics, with fast-release nanoparticle estrogen constructs effectively reducing the Bax/Bcl-2 ratio in injured tissues, indicating suppression of pro-apoptotic signaling pathways that drive neuronal loss during acute injury phases [321]. Conversely, slow-release nanoparticle estrogen constructs provided sustained therapeutic effects that prevented gliosis and penumbral demyelination in regions distal to the lesion site, addressing chronic pathological processes that limit functional recovery and axonal regeneration [321].

### 4.4. Metformin

Despite metformin’s established efficacy for spinal cord injury treatment, its therapeutic potential has been severely limited by its inability to effectively cross the blood–spinal cord barrier (BSCB), which significantly restricts drug accumulation at injury sites where therapeutic intervention is most critically needed [322]. To overcome this fundamental pharmacological limitation, researchers developed the innovative glutathione-modified macrophage-derived cell membrane-encapsulated metformin nanogels (Met-CNG-GSH), which employ biomimetic cell membrane coating technology combined with glutathione modification to enhance barrier penetration and target specificity [322]. Comprehensive pharmacokinetic analysis demonstrated that Met-CNG-GSH exhibits optimal sustained-release characteristics, providing prolonged therapeutic action that is essential for addressing the complex temporal dynamics of spinal cord injury pathophysiology [322]. The targeting efficacy of this novel delivery system was definitively confirmed through in vivo imaging studies, which revealed significant accumulation of Met-CNG-GSH at injury sites, demonstrating successful achievement of site-specific drug delivery that maximizes therapeutic concentration while minimizing systemic exposure [322]. Most importantly, animal model studies provided compelling evidence of Met-CNG-GSH’s superior therapeutic efficacy, demonstrating significant amelioration of the three primary pathological processes that drive secondary injury following spinal cord trauma: oxidative stress, neuroinflammation, and apoptotic cell death [322]. These findings establish that Met-CNG-GSH successfully transforms metformin from a systemically limited agent into a highly effective, targeted therapeutic intervention for spinal cord injury [322]. The combination of biomimetic delivery technology, sustained release kinetics, and multi-target therapeutic activity positions Met-CNG-GSH as a paradigmatic example of advanced pharmaceutical engineering that addresses both pharmacological limitations and pathophysiological complexity, offering significant promise for clinical translation and representing a new generation of precision targeted neurotherapeutics for spinal cord injury treatment [322].

### 4.5. Resveratrol

The study by Li et al. demonstrated that chitosan-modified hollow manganese dioxide nanoparticles (CM) effectively encapsulated the poorly soluble drug resveratrol (Res), resulting in a stable formulation referred to as CMR [323]. These nanoparticles had an average particle size of approximately 130 nm and achieved a drug loading efficiency of 21.39 ± 2.53% [323]. In vitro release studies showed that CMR provided a sustained release profile, with about 87% of the encapsulated Res being released gradually over a period of 36 h [323]. Therapeutically, CMR exhibited significant efficacy in spinal cord injury models [323]. It alleviated oxidative stress both at the cellular and animal levels, as evidenced by reduced levels of reactive oxygen species, malondialdehyde, and superoxide dismutase, along with increased glutathione peroxidase activity [323]. The nanoparticles also demonstrated anti-inflammatory effects, as indicated by reduced expression of inflammatory markers, such as iNOS and IL-1β via immunofluorescence, and confirmed through western blot analysis of iNOS, COX-2, IL-1β, and IL-10 levels [323]. Furthermore, CMR significantly reduced neuronal apoptosis, which was supported by the downregulation of pro-apoptotic proteins Cl caspase-3 and Bax and the upregulation of the anti-apoptotic protein Bcl-2 [323].

Resveratrol and puerarin, when co-encapsulated in polymeric nanoparticles, offer a promising therapeutic strategy for spinal cord injury, particularly by addressing the limitations of conventional systemic drug delivery [324]. This nanoparticle-based approach enhances drug stability, promotes sustained release, and effectively targets key pathological features of SCI, such as inflammation and neuronal apoptosis [324]. The resveratrol–puerarin (RES-PUE)-loaded nanoparticles exhibit favorable physicochemical properties, with an average particle size ranging from 238 to 274 nm and a mono-dispersed distribution [324]. The measured zeta potential of −12.6 ± 2.1 mV is due to the free carboxyl groups of PLGA on the nanoparticle surface, contributing to colloidal stability [324]. In terms of release kinetics, the RES-PUE formulation achieved a sustained drug release of 72–79% over 36 h, in contrast to the rapid release observed with native resveratrol and puerarin (96% and 98%, respectively, within 6 h) [324]. The optimized formulation also showed high encapsulation efficiency, reaching 74.85%, and a drug loading capacity of 15.50% at a 1:3 drug-to-polymer ratio [324]. Therapeutically, RES-PUE nanoparticles significantly reduce oxidative stress associated with ischemia–reperfusion injury, as indicated by decreased levels of malondialdehyde (MDA) and advanced oxidation protein products (AOPP) in treated rats [324]. They also modulate inflammatory responses by lowering plasma nitrite/nitrate levels and reducing p38MAPK phosphorylation while normalizing inducible nitric oxide synthase (iNOS) expression [324]. Furthermore, the nanoparticles enhance antioxidant defenses by increasing the enzymatic activity of superoxide dismutase and catalase and improving levels of reduced glutathione [324].

### 4.6. Tetramethylpyrazine

Lin et al. developed TMP-loaded nanoparticles modified with HIV trans-activator of transcription (TAT-TMP-NPs) as a targeted, sustained-release system for delivering tetramethylpyrazine (TMP) in spinal cord injury treatment [325]. These nanoparticles demonstrated favorable safety, efficient drug encapsulation, and improved pharmacokinetic and targeting profiles, making them a promising therapeutic platform for SCI [325]. Physicochemical characterization confirmed that the TAT-TMP-NPs had a uniform spherical morphology, negative surface charge (approximately −30 mV), and a stable suspension, as evidenced by their low polydispersity index (PDI < 0.3) and resistance to aggregation [325]. Particle sizes increased with drug and surface modifications, with blank nanoparticles measuring 79.07 ± 0.36 nm, TMP-NPs at 122.57 ± 2.30 nm, and TAT-TMP-NPs at 163.93 ± 0.38 nm [325]. The formulation optimized with 30 mg of TMP achieved high encapsulation efficiencies (75.54 ± 4.02% for TMP-NPs and 77.27 ± 1.99% for TAT-TMP-NPs) and comparable drug loading capacities [325]. The nanoparticles exhibited a sustained-release profile, with approximately 80–82% of TMP released over 96 h from the nanoparticle formulations, significantly outlasting the rapid 24 h release of free TMP (86%) [325]. Safety assessments showed low cytotoxicity on BV2 and SH-SY5Y cells, with cell viability above 80% even at high concentrations (1000–2000 µg/mL) and a hemolysis rate of less than 5%, confirming the system’s biocompatibility [325]. Pharmacokinetic analysis revealed that TAT-TMP-NPs improved TMP bioavailability and extended circulation time by slowing both release and elimination compared to free TMP [325]. Notably, in vivo tissue distribution studies demonstrated enhanced targeting to spinal cord tissue, particularly in SCI rats, where TMP concentrations in the spinal cord were higher than in other organs at 12 h post-administration [325].

Deng et al. indicate that the combination of an electroconductive hydrogel with tetramethylpyrazine presents a promising therapeutic strategy for spinal cord injury repair [326]. This integrated approach enhances the pharmacological effectiveness of TMP while simultaneously supporting a synergistic tissue repair environment [326]. One of the key pathological features of SCI is microvascular dysfunction, which plays a critical role in exacerbating the injury [326]. Damage to the spinal microvasculature disrupts the blood–spinal cord barrier, compromising its integrity and triggering a cascade of secondary injury processes, including inflammation, edema, and neuronal apoptosis [326]. Therefore, therapies targeting both neural regeneration and vascular stabilization—such as the TMP-loaded electroconductive hydrogel—offer a multifaceted solution to improve outcomes in SCI repair [326].

## 5. Limitations of Antioxidant Therapies in SCI

Despite extensive preclinical evidence supporting the therapeutic potential of antioxidant strategies in spinal cord injury, their clinical translation faces significant limitations that have hindered successful therapeutic applications. Single-agent antioxidant therapies have shown limited efficacy, as they fail to address the multifaceted pathophysiology of SCI, necessitating more complex combination approaches. The primary barriers to clinical translation include poor bioavailability and unfavorable pharmacokinetics of many antioxidant compounds, which limit their ability to achieve therapeutic concentrations at injury sites. Low permeability to the CNS due to the presence of physiological barriers, such as the blood–brain barrier or spinal–blood barrier, restricts the accessibility of antioxidant compounds and prevents achieving prolonged therapeutic doses. Furthermore, the heterogeneous nature of spinal cord trauma creates additional challenges for standardized treatment protocols, similar to those observed in traumatic brain injury trials. The complex temporal dynamics of oxidative stress following SCI, where excessive ROS production often overwhelms endogenous antioxidant defenses, creating a self-sustaining cycle of inflammation, apoptosis, and further ROS production, require precisely timed interventions that current delivery systems cannot adequately achieve. These limitations underscore the critical need for innovative delivery strategies, combination therapies, and personalized treatment approaches to effectively translate antioxidant-based interventions from promising preclinical results to successful clinical outcomes in SCI patients.

## 6. Conclusions

Spinal cord injury initiates a complex pathophysiological cascade in which oxidative stress is a central driver of secondary damage. Mitochondrial dysfunction, reactive oxygen and nitrogen species accumulation, and antioxidant system depletion collectively contribute to lipid peroxidation, protein and DNA damage, and neuronal apoptosis. Targeting oxidative stress thus represents a viable strategy in the development of effective treatments for SCI. In this context, antioxidants emerge as promising therapeutic agents. Experimental evidence supports the neuroprotective efficacy of several natural and synthetic compounds through mechanisms including inhibition of oxidative stress, modulation of inflammatory and apoptotic pathways, and enhancement of neurotrophic signaling. These interventions show potential for preserving spinal cord tissue and improving functional outcomes, particularly when matched to injury severity and delivered with precision.

The field of antioxidant therapeutics for spinal cord injury stands at a critical juncture where promising preclinical findings must be translated into effective clinical interventions. This limitation has prompted a shift toward combination strategies, which leverage the synergistic effects of multiple antioxidants, moving beyond the historical focus on single-agent therapies that have demonstrated limited clinical efficacy. Future research directions must prioritize the development of targeted delivery systems to overcome existing pharmacokinetic limitations. Advanced delivery systems, such as nanoparticles, have been developed to improve the stability and efficacy of antioxidant compounds. Future advancements may include the use of intrathecal catheters, which allow for the steady and repeated infusion of antioxidants to the injury site, potentially enhancing controlled and targeted interventions. The integration of molecular approaches represents another promising avenue for advancement. miRNAs play a crucial role in modulating the oxidative stress response across various disease models and have been identified as key regulators of the antioxidant response by targeting genes encoding antioxidant enzymes. Critical research gaps that require urgent attention include establishing optimal therapeutic windows, understanding cell-type-specific responses to oxidative stress, and developing standardized protocols for complex therapeutic combinations. The molecular mechanisms and signaling pathways underlying the therapeutic effects of EVs in SCI remain largely unexplored. The development of sophisticated experimental models will be essential for advancing the field. Spinal cord organoids derived from iPSCs provide a physiologically relevant in vitro model to study neural networks and cellular interactions within the spinal cord, as well as to screen experimental therapies. Successful clinical translation will ultimately depend on addressing regulatory challenges, establishing comprehensive safety profiles for novel therapeutic combinations, and developing evidence-based treatment protocols that can be seamlessly integrated into current clinical practice to improve outcomes for patients with spinal cord injuries.

## Figures and Tables

**Table 1:** Mechanisms of oxidative stress in spinal cord injury.

Category	Mechanism/Source	Key Components	Molecular Details	Consequences
PRIMARY ROS GENERATION	Mitochondrial Dysfunction	Electron transport chain disruption Complexes I and III electron leakage Compromised energy metabolism	Superoxide (O_2_^−^•)—one-electron reduction H_2_O_2_—two-electron transfer Hydroxyl radical (•OH) Singlet oxygen (^1^O_2_)	Self-perpetuating cycle of ROS production and mitochondrial damage
	Fenton Chemistry	Transition metal catalysis Iron from hemoglobin and ferritin BSCB breakdown	Fe^2+^/Cu^+^ + H_2_O_2_ → •OH + OH^−^ + Fe^3+^/Cu^2+^ Highly reactive hydroxyl radicals	Enhanced lipid peroxidation and cellular damage
	Energy Crisis	ATP depletion Coenzyme Q10 depletion Cytochrome c loss	Na^+^/K^+^-ATPase failure Ca^2+^-ATPase dysfunction Mitochondrial membrane potential disruption	Ionic imbalances Calcium overload
	Permeability Transition	Ca^2+^ overload trigger Pore formation Pro-apoptotic factor release	Cytochrome c release Phospholipase activation Protease activation Endonuclease activation	Apoptotic cell death pathway activation
CELLULAR ROS SOURCES	NADPH Oxidase	Activated microglia Infiltrating macrophages Inflammatory response	Superoxide generation Dual role: neuroprotection/neurodegeneration Controlled vs. excessive production	Tissue damage and secondary injury propagation
	Neutrophil Infiltration	Early infiltration (hours) Myeloperoxidase activity Respiratory burst	Hypochlorous acid (HOCl) Chlorinated oxidants Massive superoxide and H_2_O_2_ production	Significant contribution to oxidative stress
	Xanthine Oxidase	Ischemia–reperfusion injury Dehydrogenase → oxidase conversion ATP breakdown products	Hypoxanthine metabolism Xanthine metabolism Superoxide production during purine catabolism	Additional ROS burden during reperfusion
DAMAGE MECHANISMS	Lipid Peroxidation	PUFA in membranes Chain reaction propagation Iron-catalyzed process	•OH abstracts H atoms from PUFA 4-hydroxynonenal (4-HNE) Malondialdehyde (MDA) Covalent protein modification	Membrane disruption Neural tissue vulnerability
	Protein Oxidation	Amino acid modification Cysteine/methionine targets Antioxidant enzyme loss	Disulfide bond formation Methionine sulfoxide Protein carbonyls Protein aggregation	Enzyme inactivation Cellular dysfunction
	DNA Oxidation	Base modification Strand breaks mtDNA vulnerability	8-hydroxyguanosine Sugar–phosphate backbone damage Limited mtDNA repair mechanisms Mutation accumulation	Genomic instability Cell death pathway activation
SECONDARY CASCADES	Peroxynitrite Formation	NO + O_2_^−^• combination Diffusion-limited reaction Highly reactive species	NO + O_2_^−^• → ONOO^−^ Protein nitrosylation Lipid oxidation at diffusion limits DNA damage	Potent oxidative and nitrosative damage
	PARP Activation	DNA damage response Energy depletion NAD^+^ consumption	Poly(ADP-ribose) polymerase activation NAD^+^ and ATP depletion Cell death promotion Additional oxidative stress source	Energy crisis Cell death acceleration
ANTIOXIDANT DEPLETION	Enzymatic Systems	Direct oxidative modification Transcriptional downregulation Nrf2-ARE pathway disruption	SOD (superoxide dismutase) Catalase Glutathione peroxidase (GPx) Glutathione reductase	Compromised cellular defense Vicious cycle formation
	Non-enzymatic Systems	Rapid consumption post-injury NADPH limitation Impaired recycling	GSH (glutathione) Ascorbic acid α-tocopherol Coenzyme Q10	Primary defense depletion Oxidative stress amplification
SIGNAL TRANSDUCTION	JNK Pathway	ROS accumulation response Mitochondrial dysfunction Pro-apoptotic activation	c-Jun N-terminal kinase Pro-apoptotic protein phosphorylation Transcription factor activation	Apoptotic cell death promotion
	p38 MAPK	Oxidative stress activation Inflammatory gene expression Cytokine production	p38 mitogen-activated protein kinase Inflammatory mediator transcription Additional ROS generation	Tissue damage propagation and inflammation
	NF-κB Pathway	ROS-mediated activation Dual protective/harmful role Transcriptional regulation	Nuclear factor-κB Inflammatory mediator transcription Antioxidant gene upregulation Complex dual nature	Inflammation with some protective effects
	Nrf2-ARE System	Antioxidant response disruption Transcriptional downregulation Protective enzyme synthesis	Nuclear factor erythroid 2-related factor 2 Antioxidant response elements Protective enzyme transcription	Compromised antioxidant defense synthesis

## Data Availability

No new data were created in this study.
